# Supplementary material for: Temporal Splicing Switches in Elements of the TNF-Pathway Identified by Computational Analysis of Transcriptome Data for Human Cell Lines
Source: Int J Mol Sci. 2019 Mar 8;20(5):1182. doi: 10.3390/ijms20051182 (PMC6429354; doi:10.3390/ijms20051182)
Supplement: Supplementary file 1 [file ijms-20-01182-s001.zip › Genovetal_Figure_3_13.02.2019.pdf]

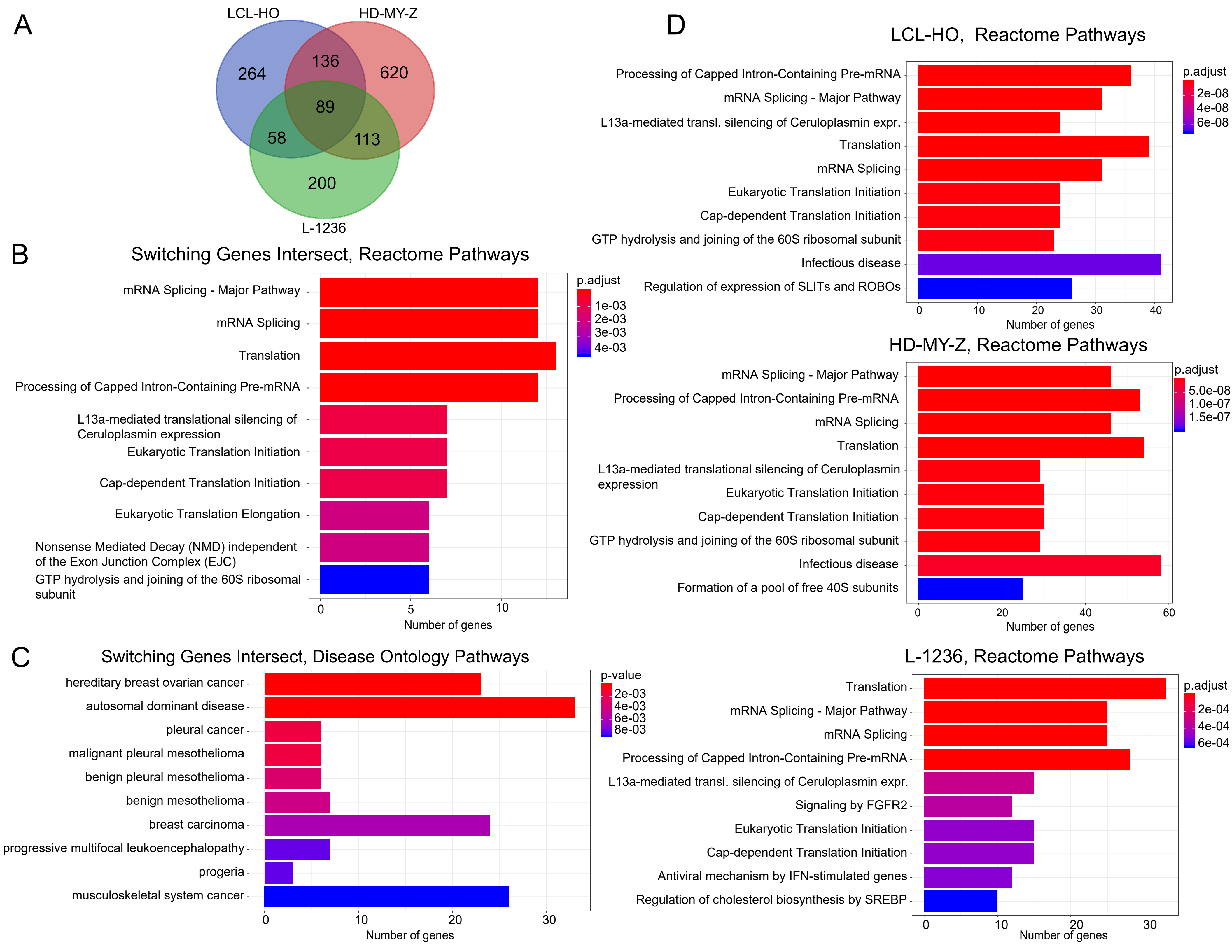
A
LCL-HO
HD-MY-Z
L-1236
264
136
620
89
58
113
200
B
Switching Genes Intersect, Reactome Pathways
mRNA Splicing - Major Pathway
mRNA Splicing
Translation
Processing of Capped Intron-Containing Pre-mRNA
L13a-mediated translational silencing of Ceruloplasmin expression
Eukaryotic Translation Initiation
Cap-dependent Translation Initiation
Eukaryotic Translation Elongation
Nonsense Mediated Decay (NMD) independent of the Exon Junction Complex (EJC)
GTP hydrolysis and joining of the 60S ribosomal subunit
p.adjust
1e-03
2e-03
3e-03
4e-03
0
5
10
Number of genes
C
Switching Genes Intersect, Disease Ontology Pathways
hereditary breast ovarian cancer
autosomal dominant disease
pleural cancer
malignant pleural mesothelioma
benign pleural mesothelioma
benign mesothelioma
breast carcinoma
progressive multifocal leukoencephalopathy
progeria
musculoskeletal system cancer
p-value
2e-03
4e-03
6e-03
8e-03
0
10
20
30
Number of genes
D
LCL-HO, Reactome Pathways
Processing of Capped Intron-Containing Pre-mRNA
mRNA Splicing - Major Pathway
L13a-mediated transl. silencing of Ceruloplasmin expr.
Translation
mRNA Splicing
Eukaryotic Translation Initiation
Cap-dependent Translation Initiation
GTP hydrolysis and joining of the 60S ribosomal subunit
Infectious disease
Regulation of expression of SLITs and ROBOs
p.adjust
2e-08
4e-08
6e-08
0
10
20
30
40
Number of genes
HD-MY-Z, Reactome Pathways
mRNA Splicing - Major Pathway
Processing of Capped Intron-Containing Pre-mRNA
mRNA Splicing
Translation
L13a-mediated translational silencing of Ceruloplasmin expression
Eukaryotic Translation Initiation
Cap-dependent Translation Initiation
GTP hydrolysis and joining of the 60S ribosomal subunit
Infectious disease
Formation of a pool of free 40S subunits
p.adjust
5.0e-08
1.0e-07
1.5e-07
0
20
40
60
Number of genes
L-1236, Reactome Pathways
Translation
mRNA Splicing - Major Pathway
mRNA Splicing
Processing of Capped Intron-Containing Pre-mRNA
L13a-mediated transl. silencing of Ceruloplasmin expr.
Signaling by FGFR2
Eukaryotic Translation Initiation
Cap-dependent Translation Initiation
Antiviral mechanism by IFN-stimulated genes
Regulation of cholesterol biosynthesis by SREBP
p.adjust
2e-04
4e-04
6e-04
0
10
20
30
Number of genes
