# Supplementary material for: Temporal Splicing Switches in Elements of the TNF-Pathway Identified by Computational Analysis of Transcriptome Data for Human Cell Lines
Source: Int J Mol Sci. 2019 Mar 8;20(5):1182. doi: 10.3390/ijms20051182 (PMC6429354; doi:10.3390/ijms20051182)
Supplement: Supplementary file 1 [file ijms-20-01182-s001.zip › Genovetal_Figure_S6_13.02.2019.pdf]

LCL-HO

A

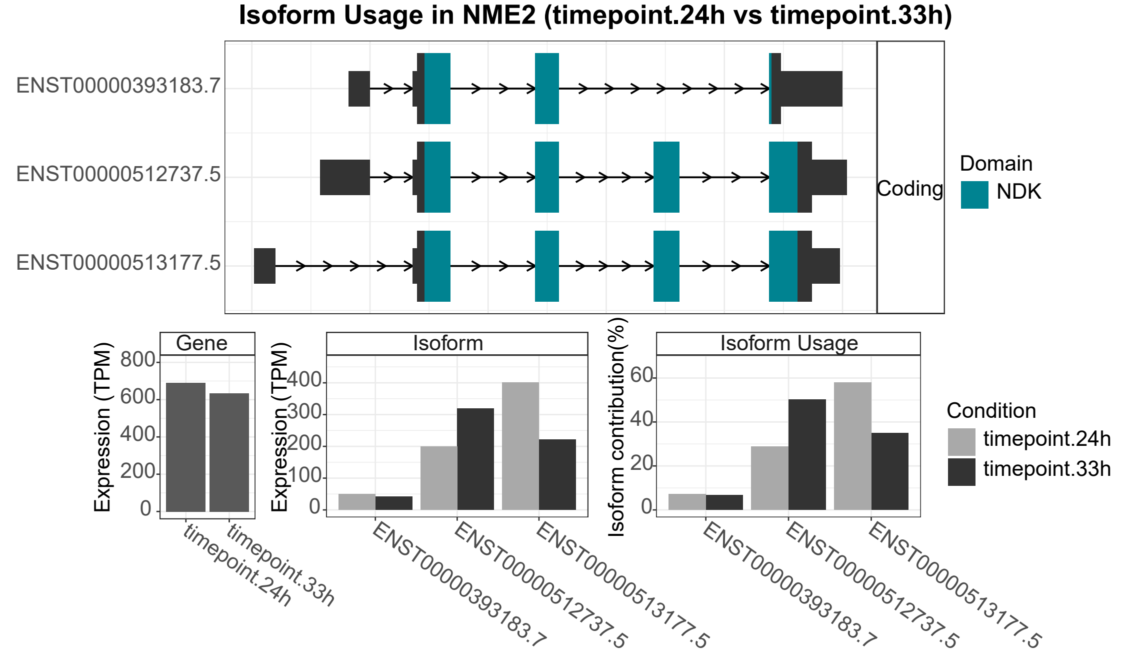
Isoform Usage in NME2 (timepoint.24h vs timepoint.33h)
ENST00000393183.7
ENST00000512737.5
ENST00000513177.5
Coding
Domain
NDK
Gene
Expression (TPM)
800
600
400
200
0
timepoint.24h
timepoint.33h
Isoform
Expression (TPM)
400
300
200
100
0
Isoform Usage
Isoform contribution(%)
60
40
20
0
Condition
timepoint.24h
timepoint.33h

B

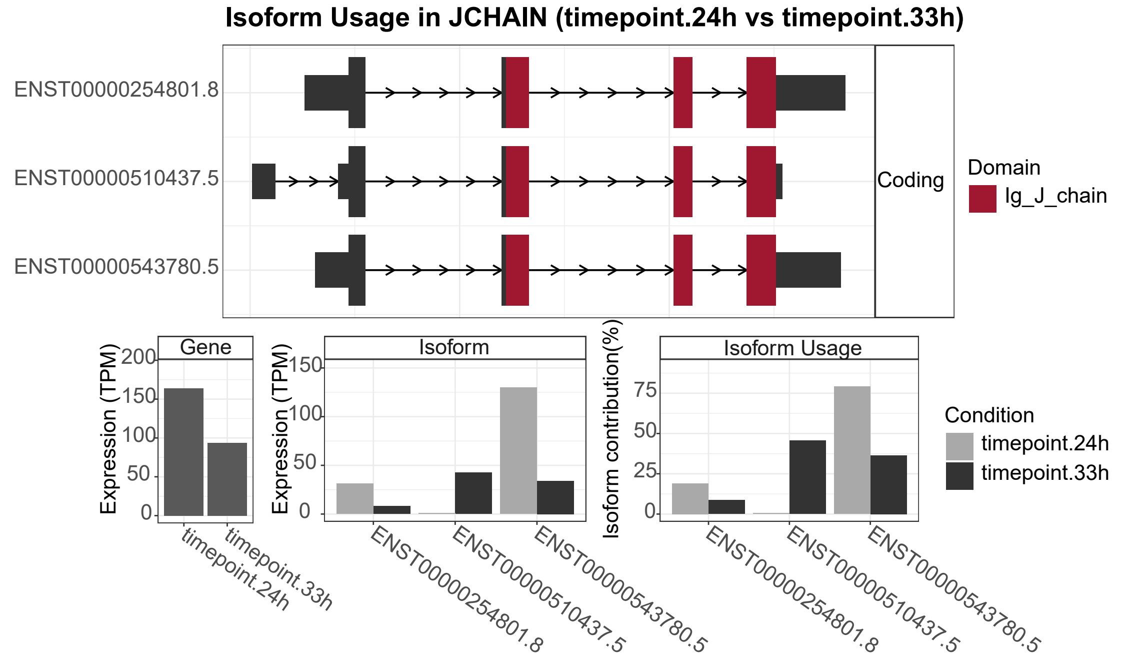
Isoform Usage in JCHAIN (timepoint.24h vs timepoint.33h)
ENST00000254801.8
ENST00000510437.5
ENST00000543780.5
Coding
Domain
Ig_J_chain
Gene
Expression (TPM)
200
150
100
50
0
timepoint.24h
timepoint.33h
Isoform
Expression (TPM)
150
100
50
0
Isoform Usage
Isoform contribution(%)
75
50
25
0
Condition
timepoint.24h
timepoint.33h
