# Supplementary figures and images for: Temporal Splicing Switches in Elements of the TNF-Pathway Identified by Computational Analysis of Transcriptome Data for Human Cell Lines
Source: Int J Mol Sci. 2019 Mar 8;20(5):1182. doi: 10.3390/ijms20051182 (PMC6429354; doi:10.3390/ijms20051182)

A

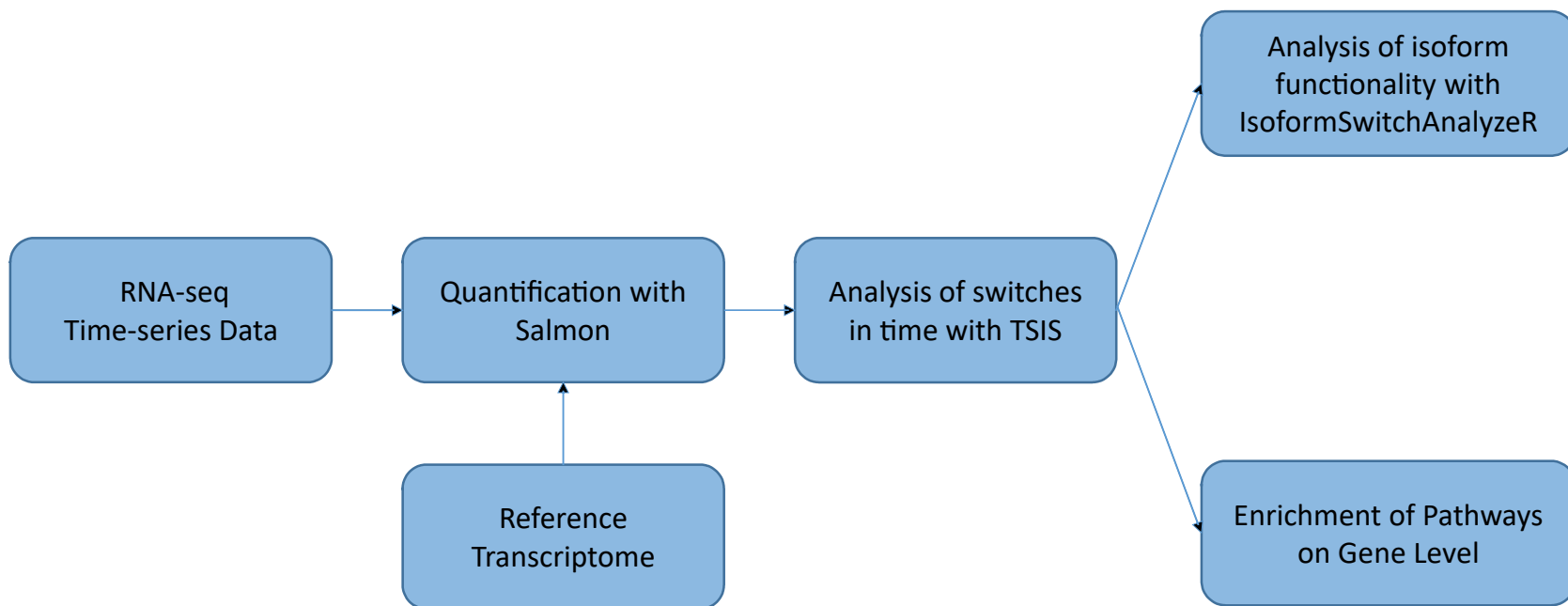

B

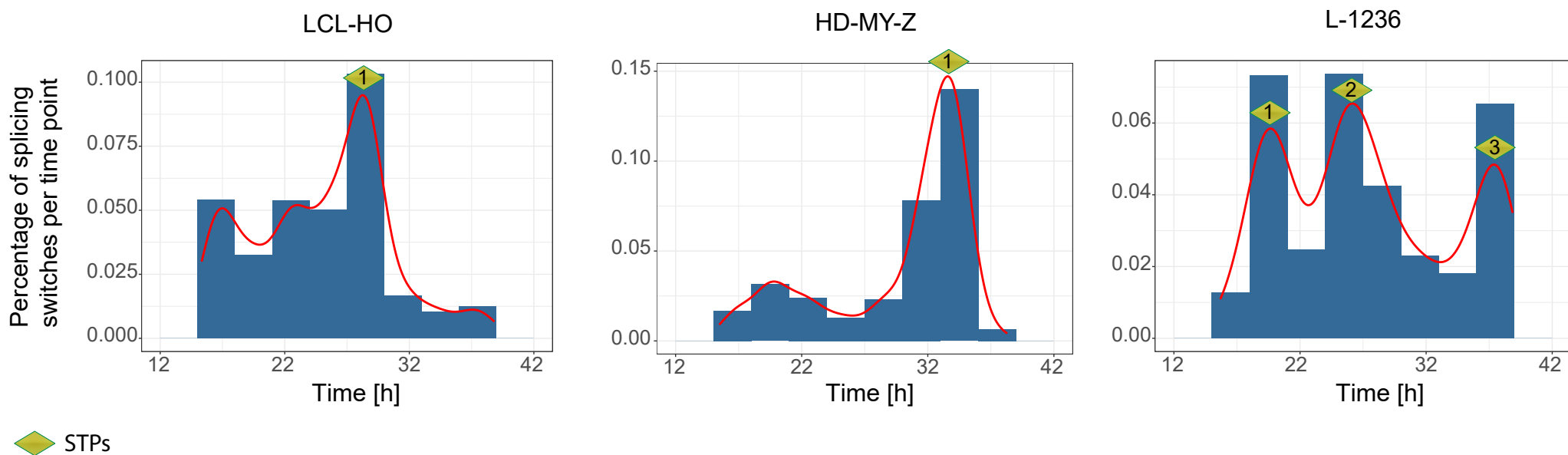

Supplement: Supplementary file 1 [file ijms-20-01182-s001.zip › Genovetal_Figure_1_13.02.2019.pdf]

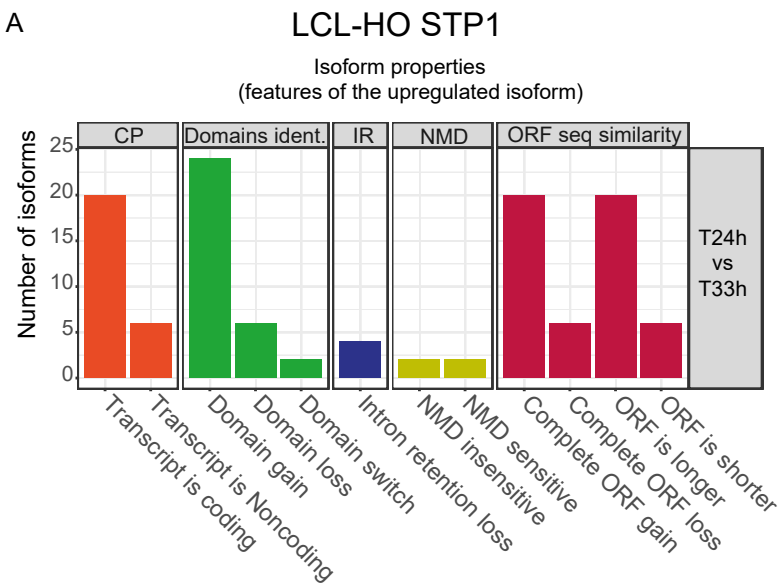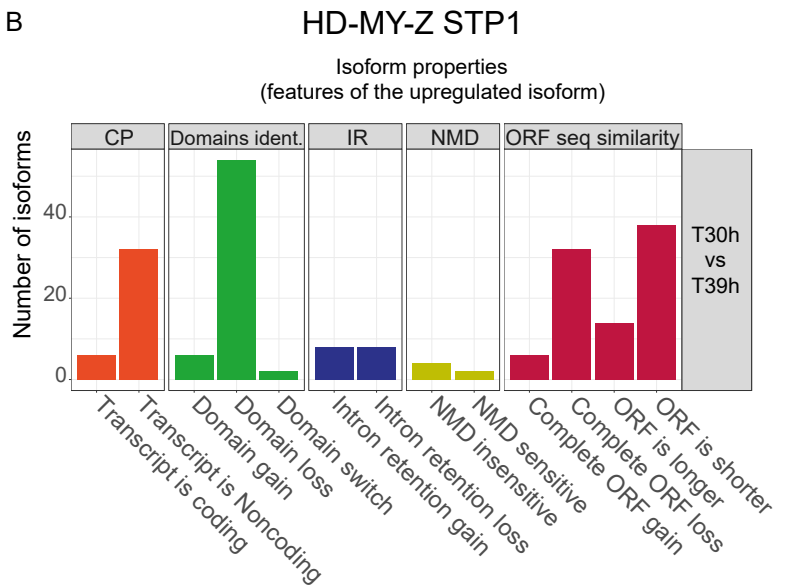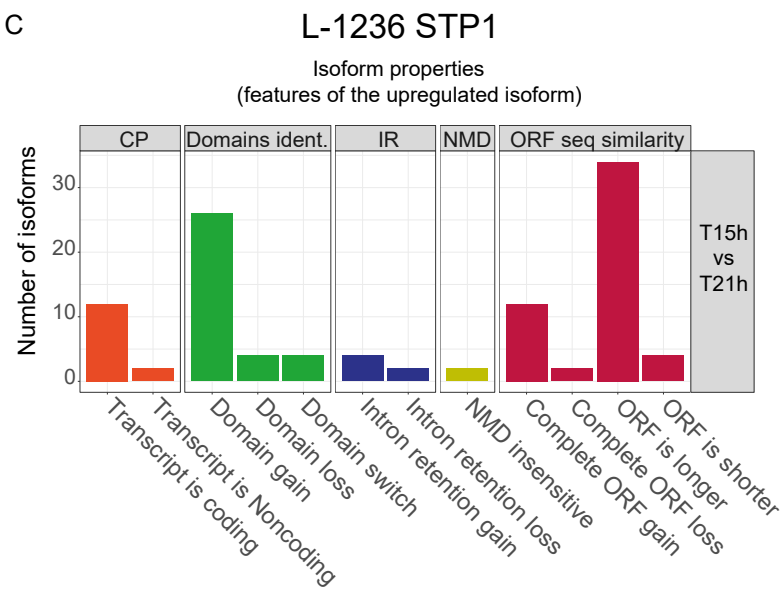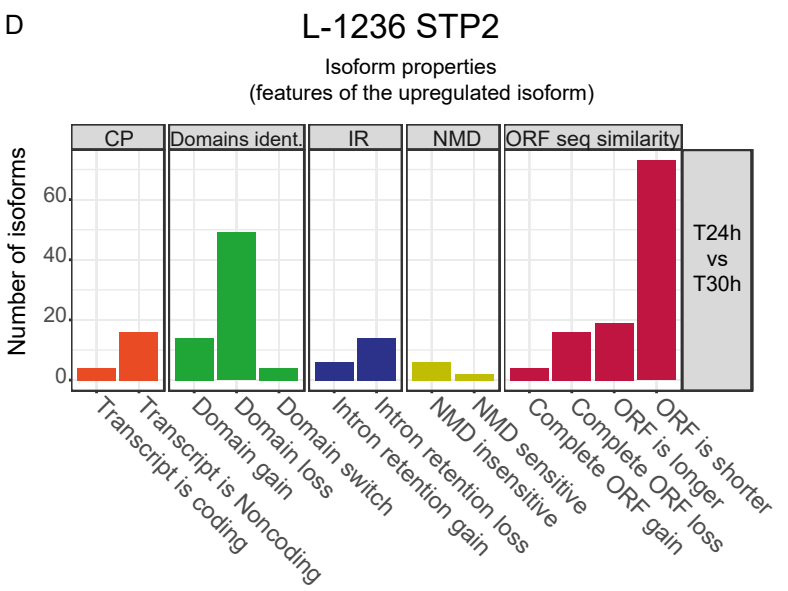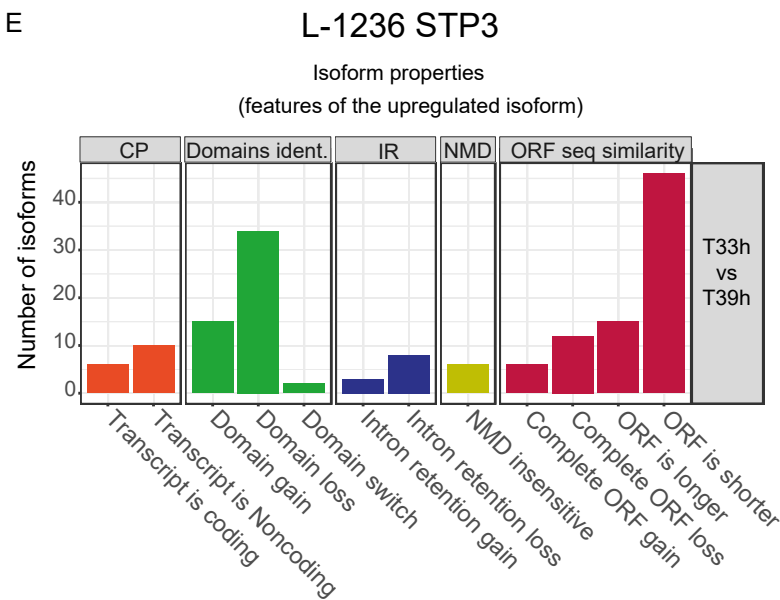

Supplement: Supplementary file 1 [file ijms-20-01182-s001.zip › Genovetal_Figure_2_13.02.2019.pdf]

**A****LCL-HO**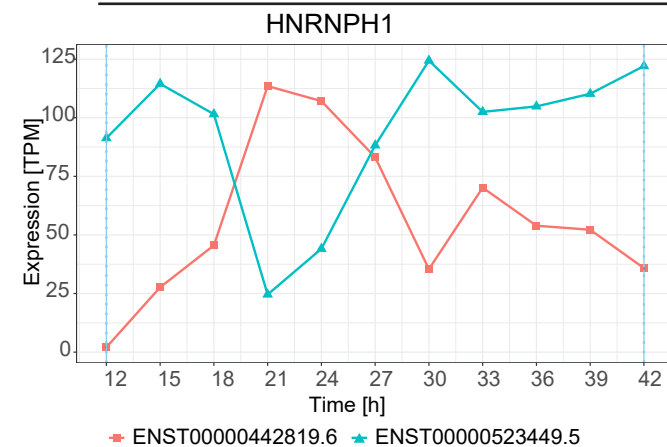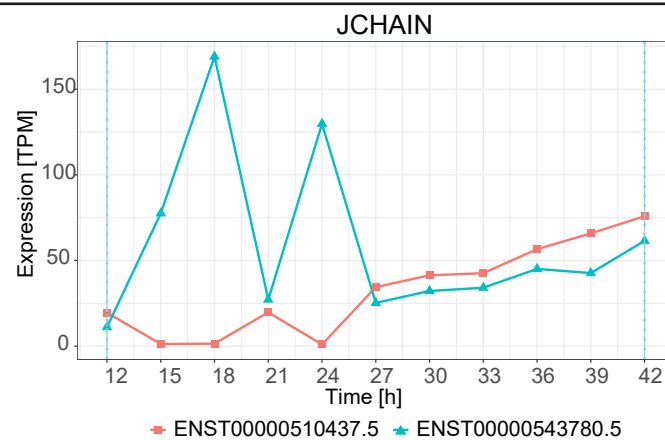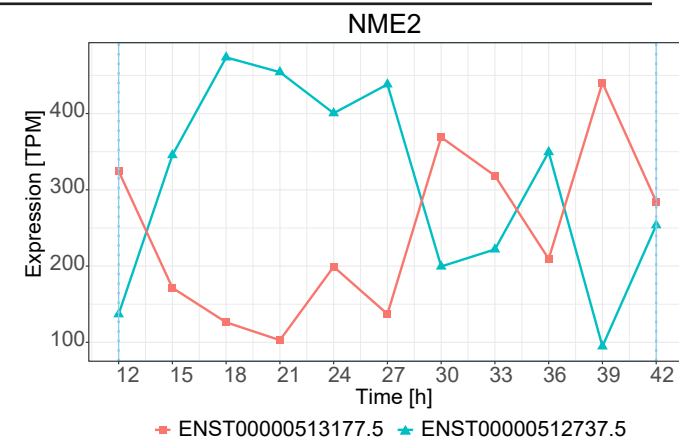**B****HD-MY-Z**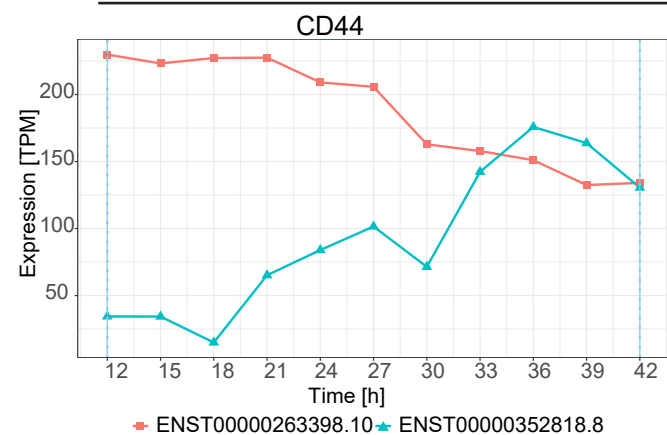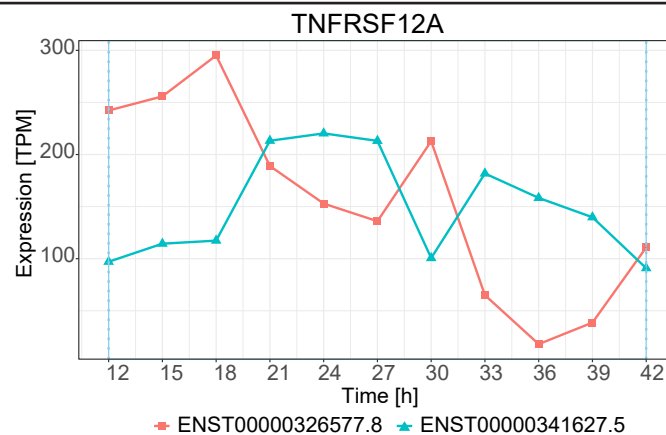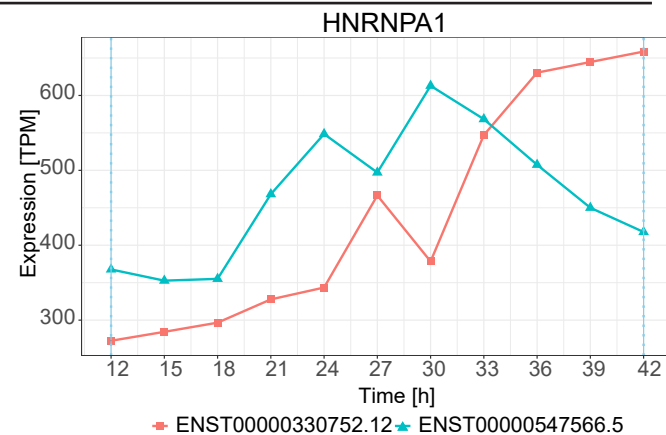**C****L-1236**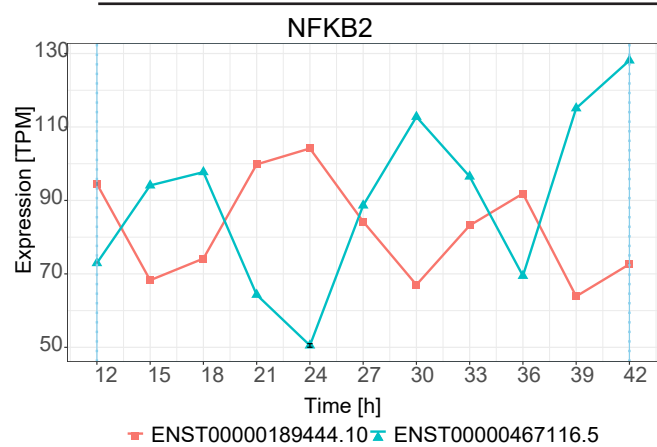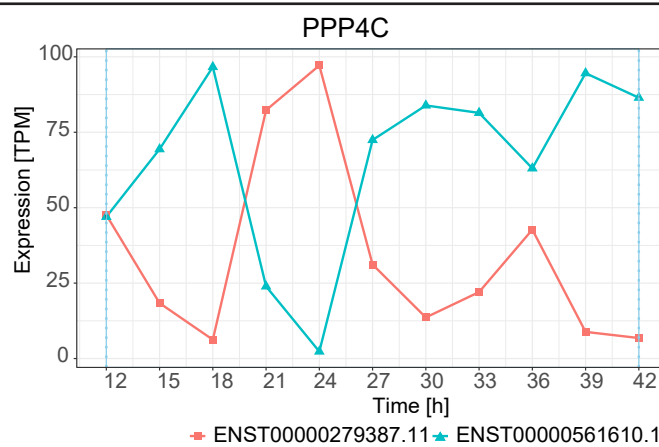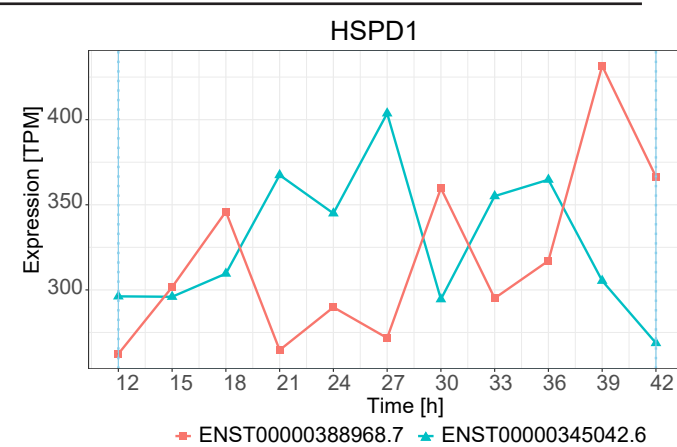

Supplement: Supplementary file 1 [file ijms-20-01182-s001.zip › Genovetal_Figure_4_13.02.2019.pdf]

A

LCL-HO

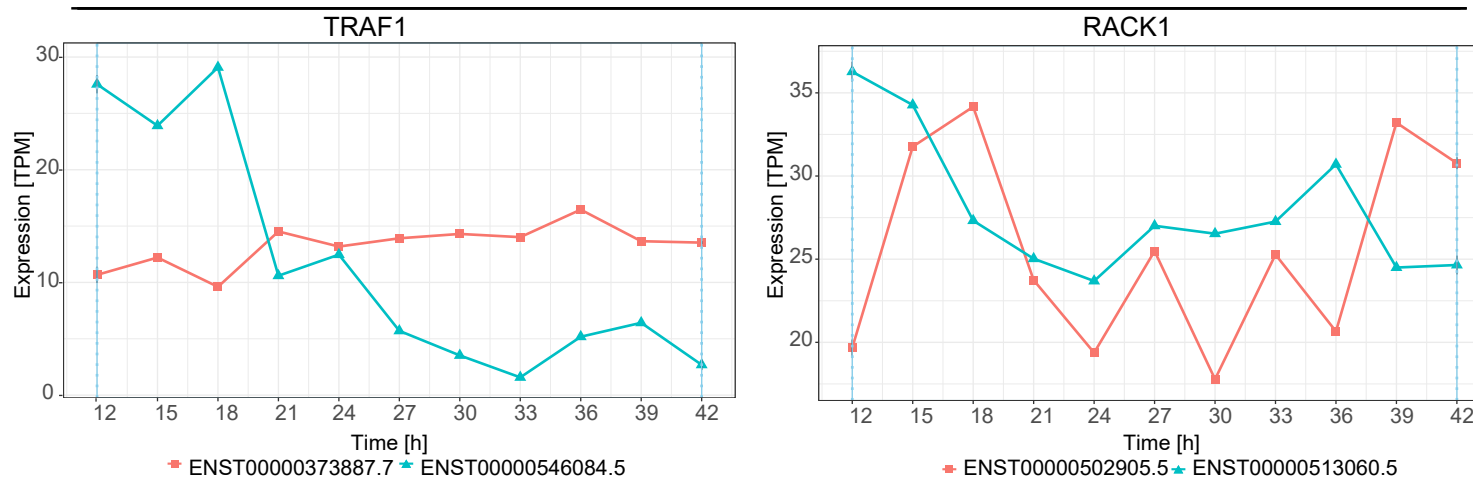

B

HD-MY-Z

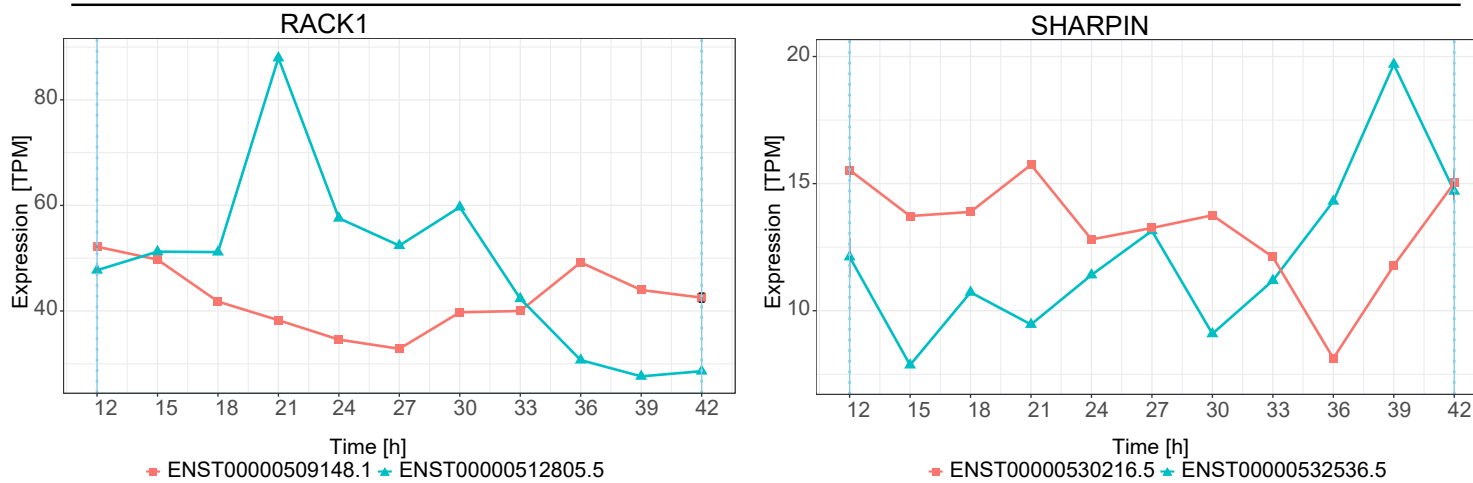

C

L-1236

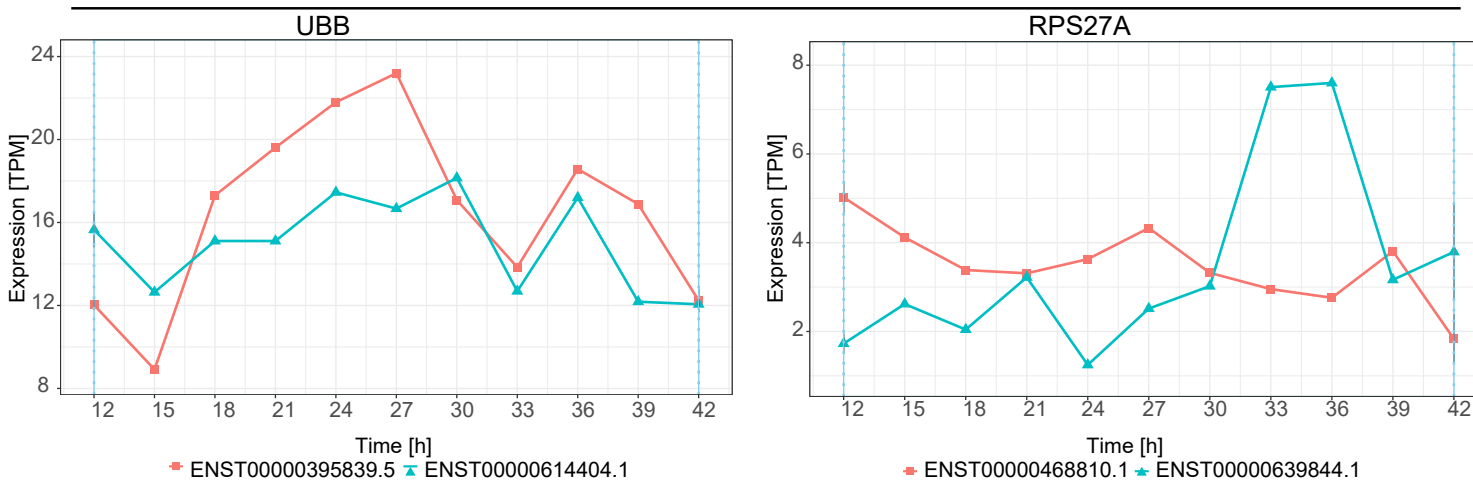

Supplement: Supplementary file 1 [file ijms-20-01182-s001.zip › Genovetal_Figure_5_13.02.2019.pdf]

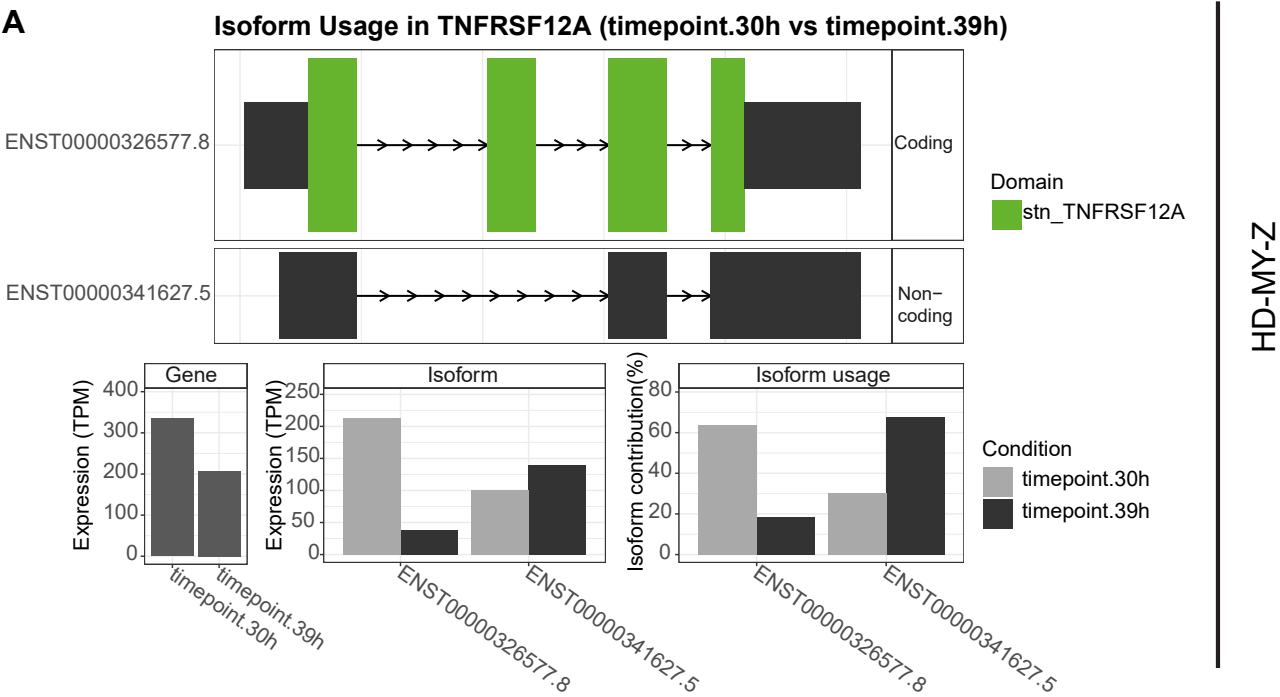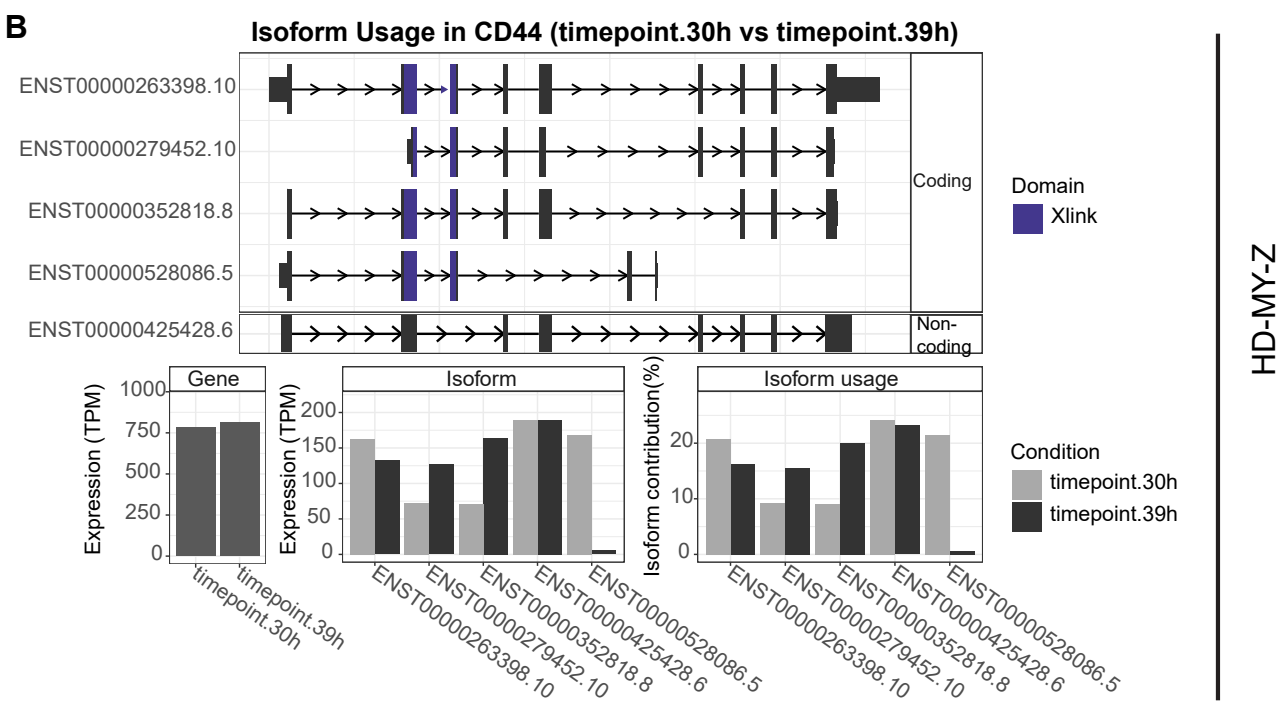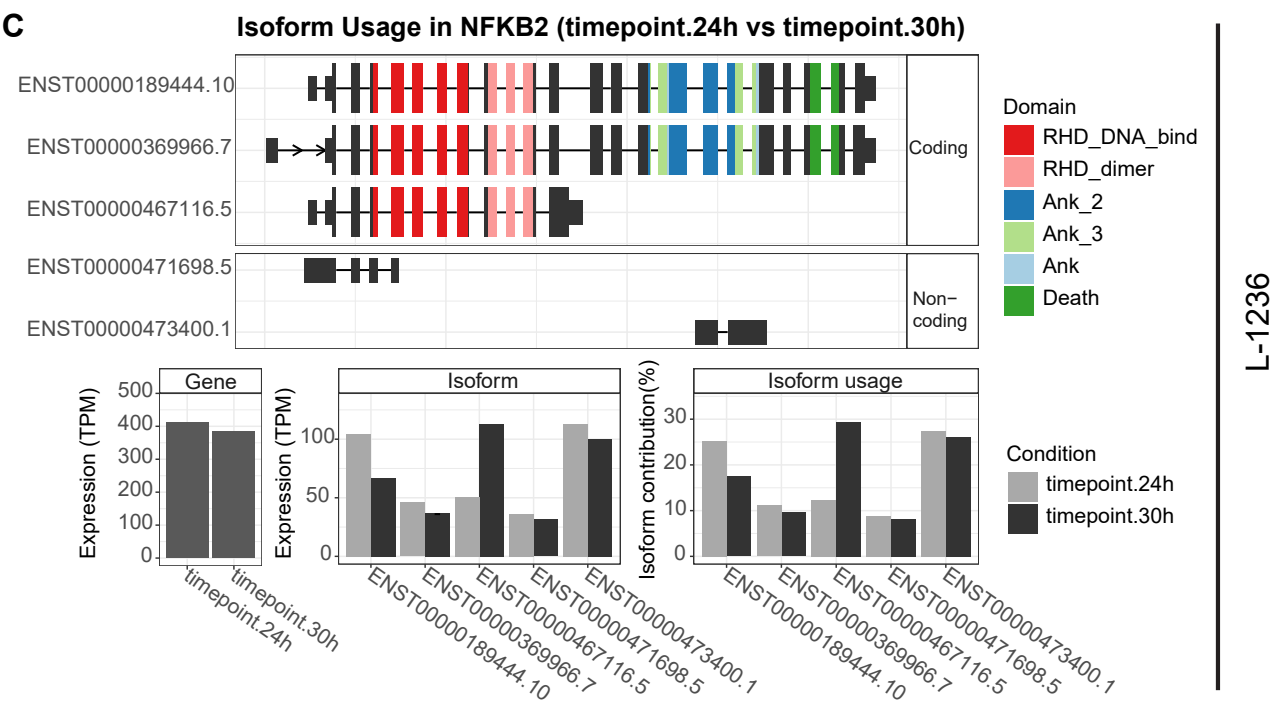

Supplement: Supplementary file 1 [file ijms-20-01182-s001.zip › Genovetal_Figure_6_13.02.2019.pdf]

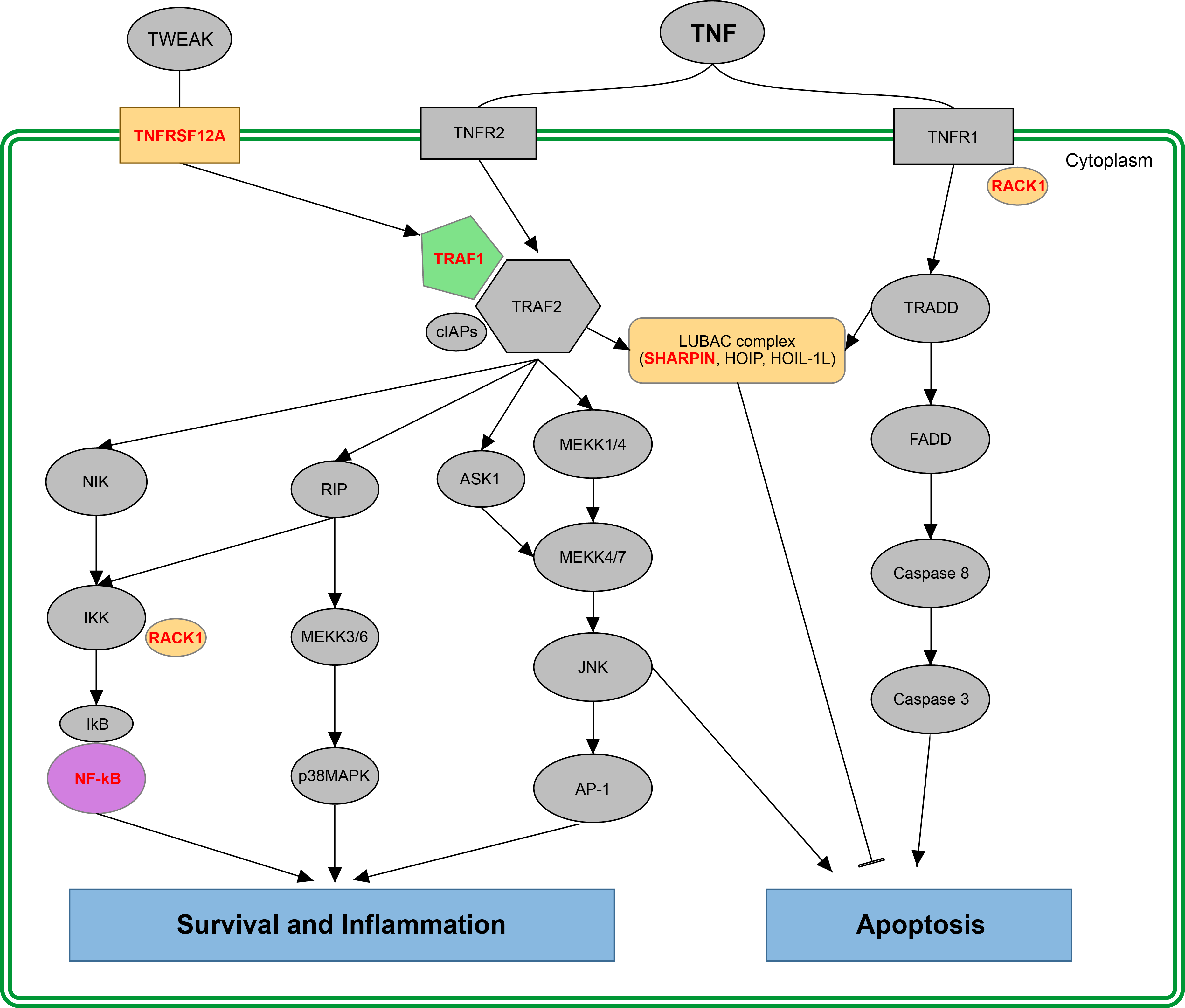

Supplement: Supplementary file 1 [file ijms-20-01182-s001.zip › Genovetal_Figure_7_13.02.2019.png]

LCL-HO STP1

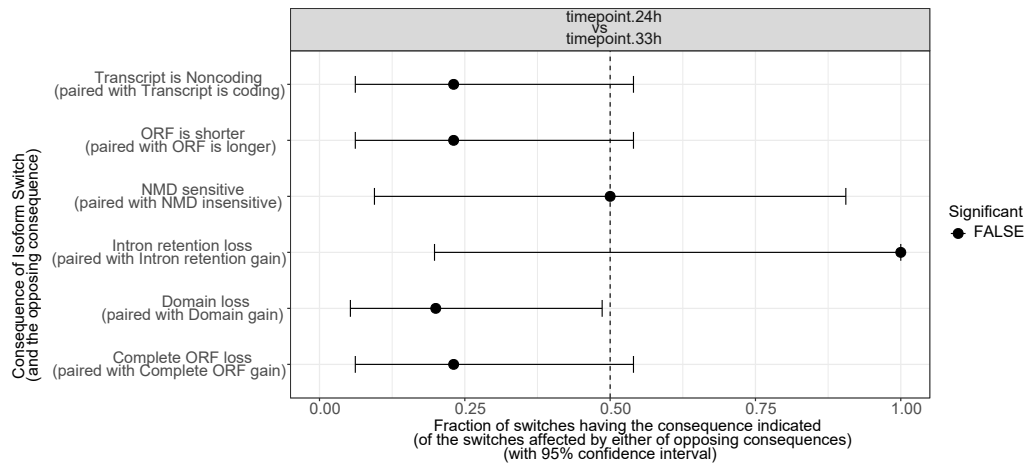

HD-MY-Z STP1

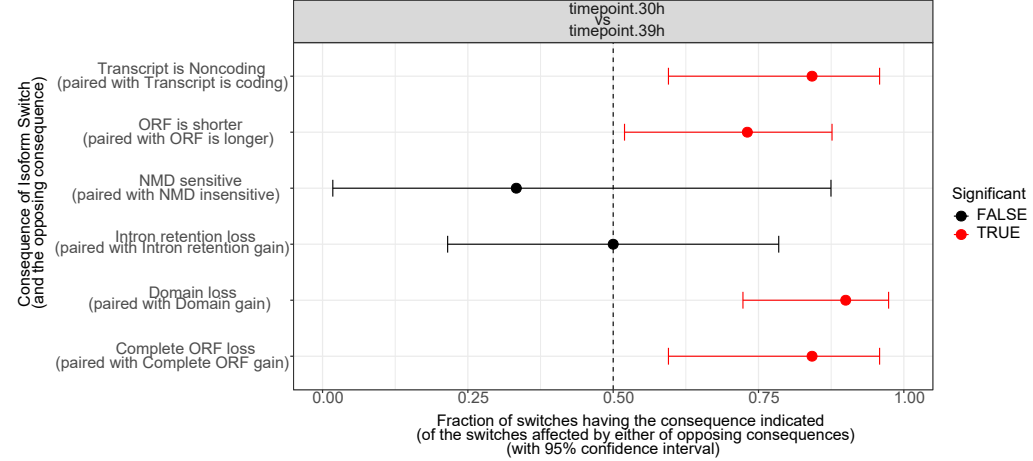

L-1236 STP1

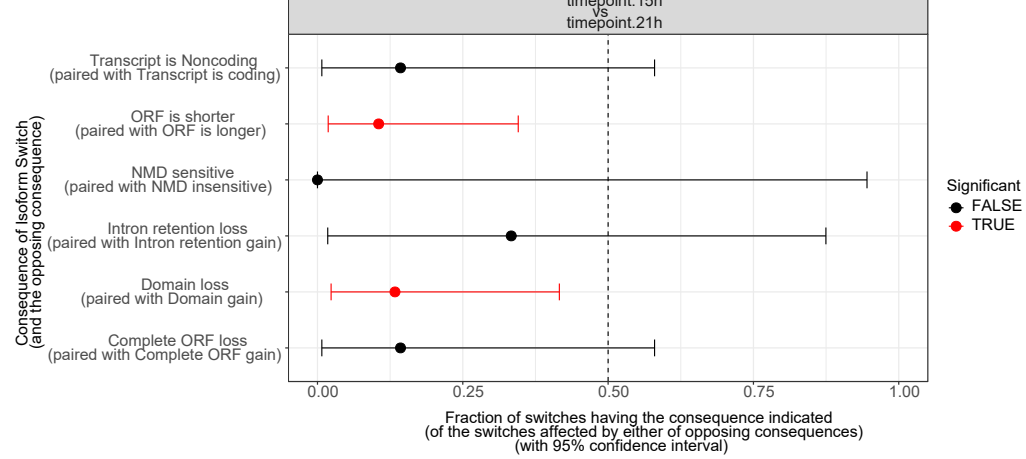

L-1236 STP2

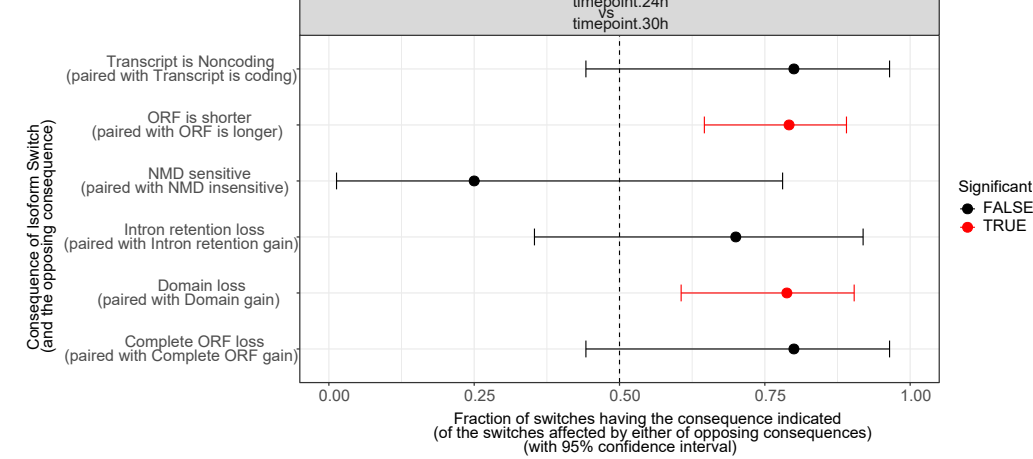

L-1236 STP3

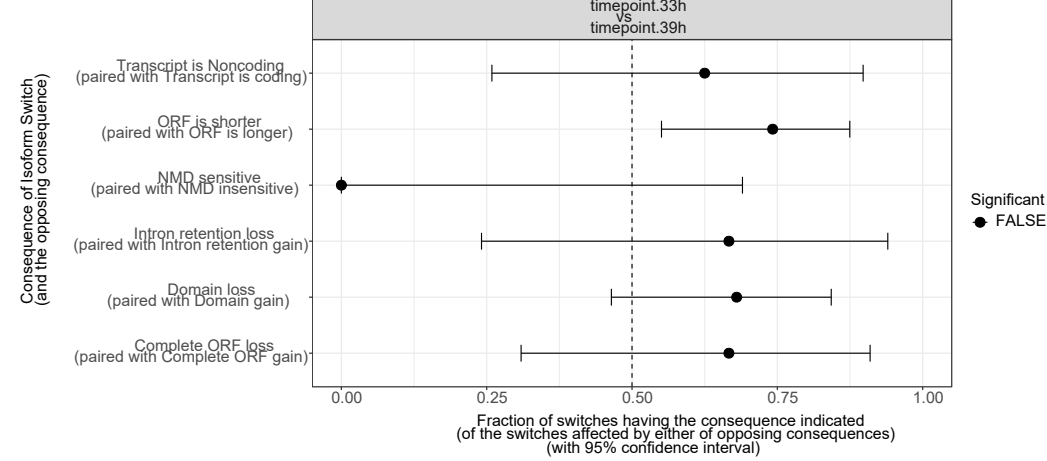

Supplement: Supplementary file 1 [file ijms-20-01182-s001.zip › Genovetal_Figure_S1_13.02.2019.pdf]

A

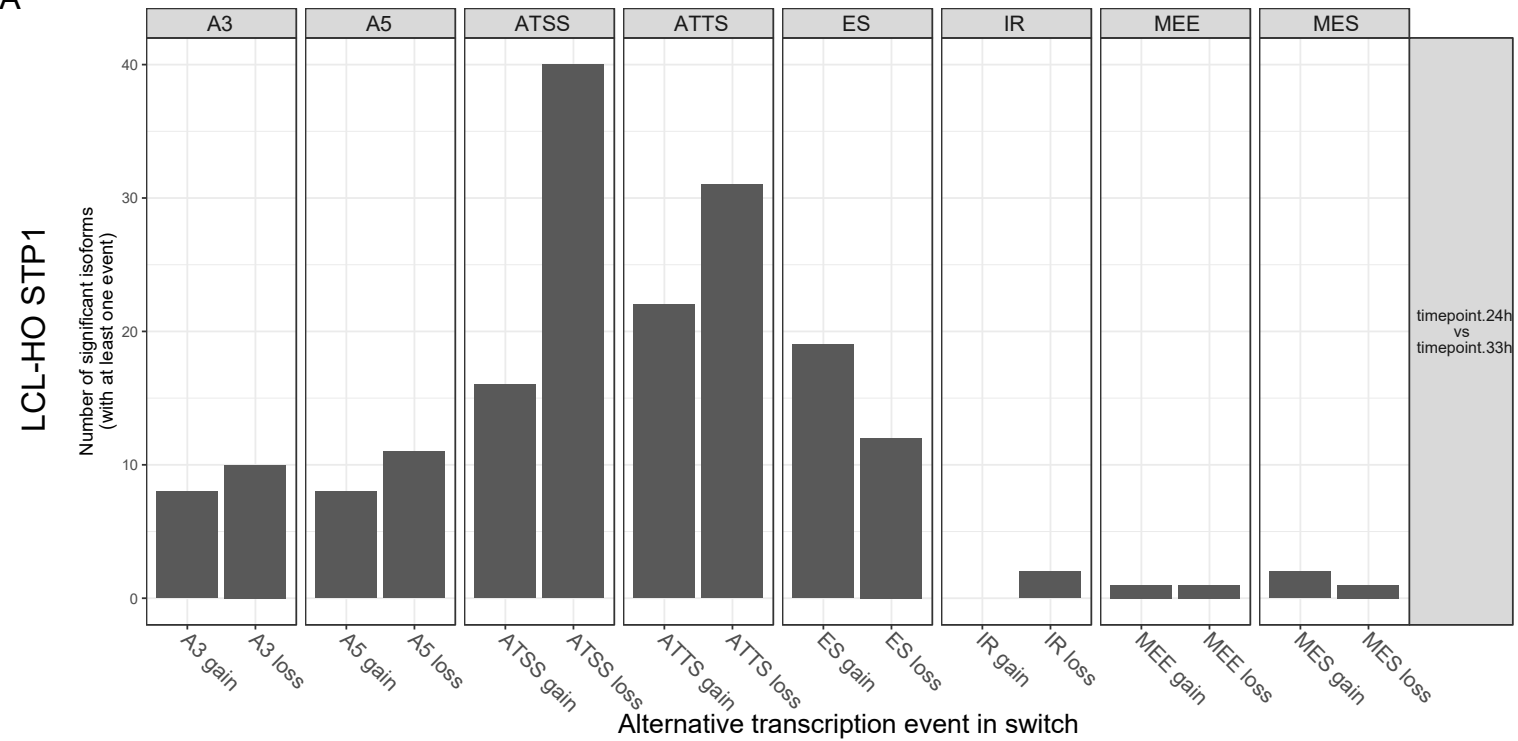

B

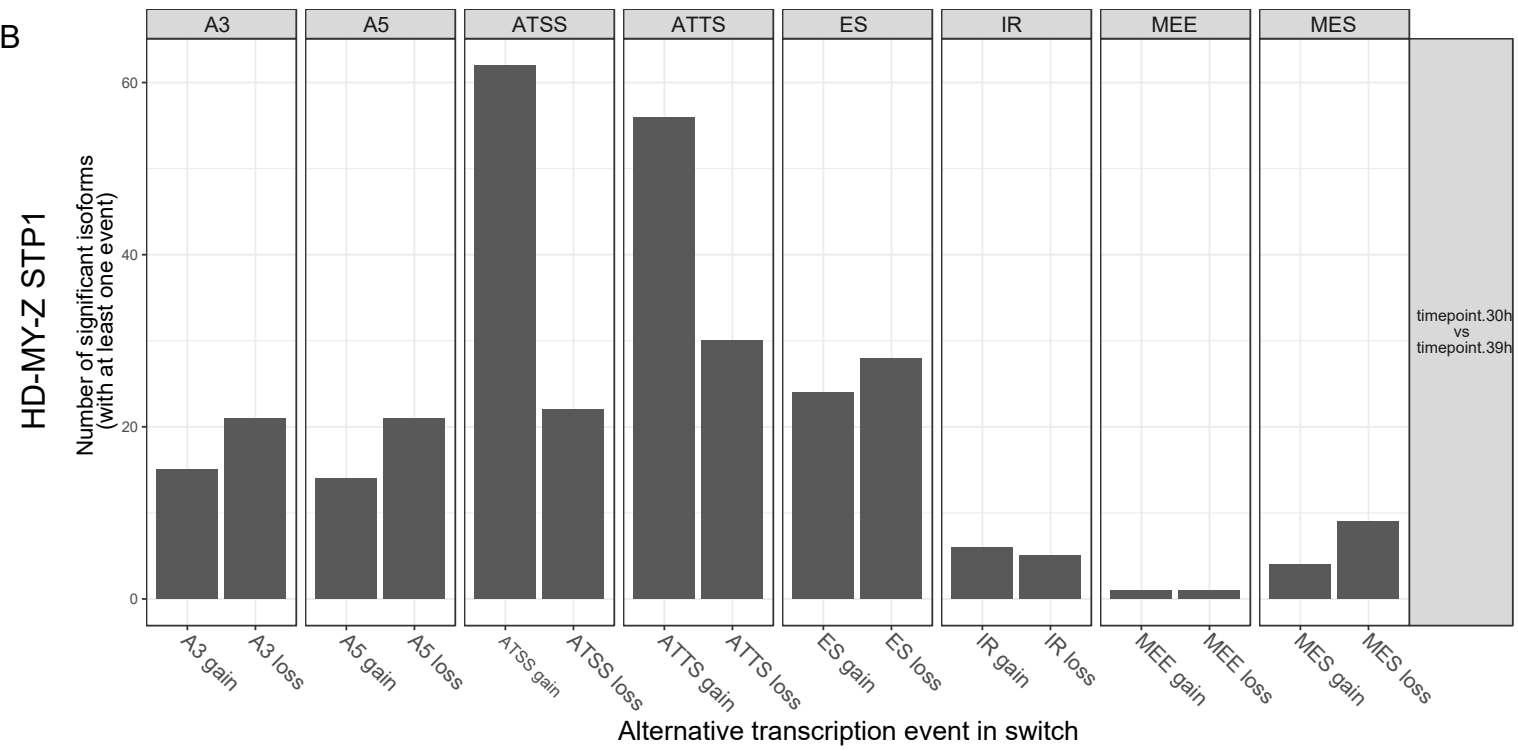

Supplement: Supplementary file 1 [file ijms-20-01182-s001.zip › Genovetal_Figure_S2_13.02.2019.pdf]

A

L-1236 STP1

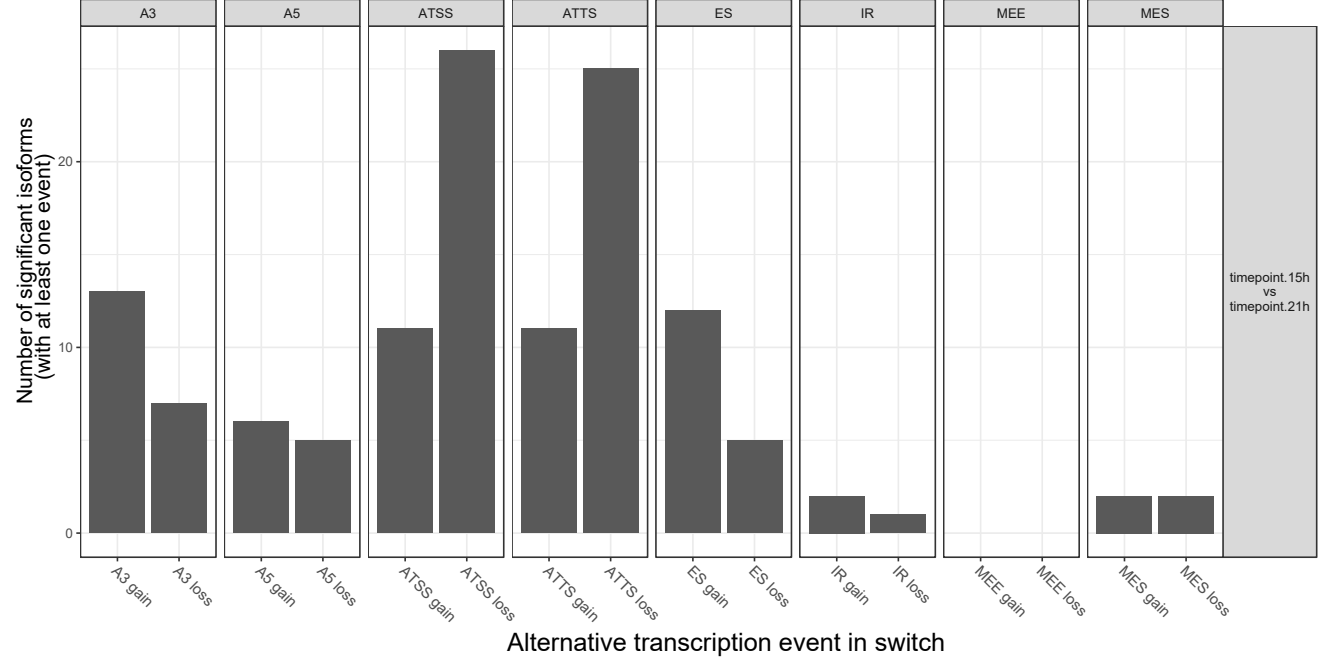

B

L-1236 STP2

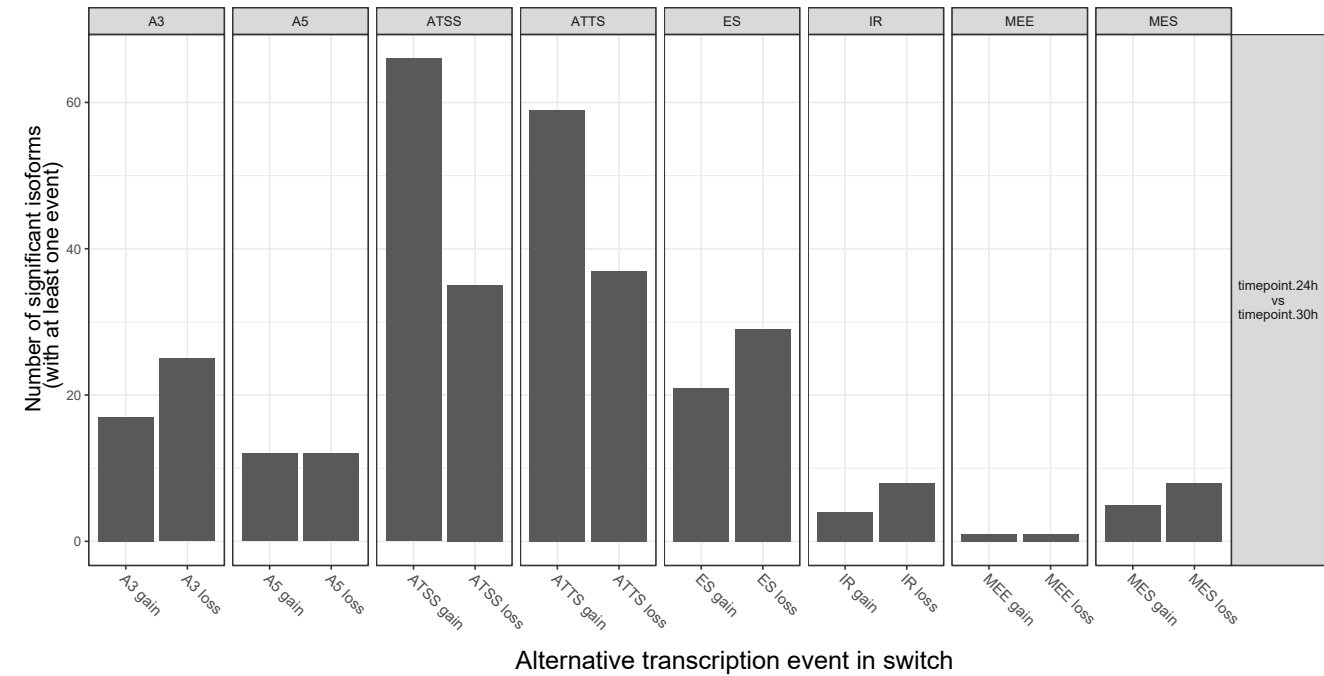

C

L-1236 STP3

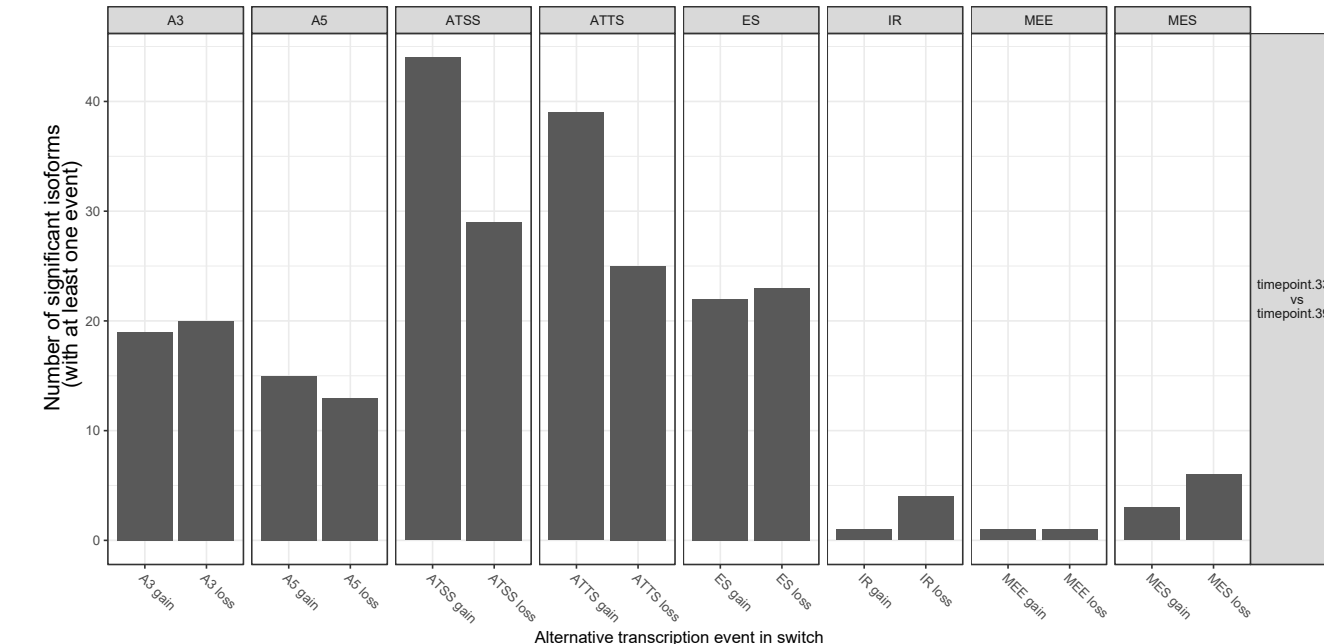

Supplement: Supplementary file 1 [file ijms-20-01182-s001.zip › Genovetal_Figure_S3_13.02.2019.pdf]

A

## LCL-HO, unique switching genes

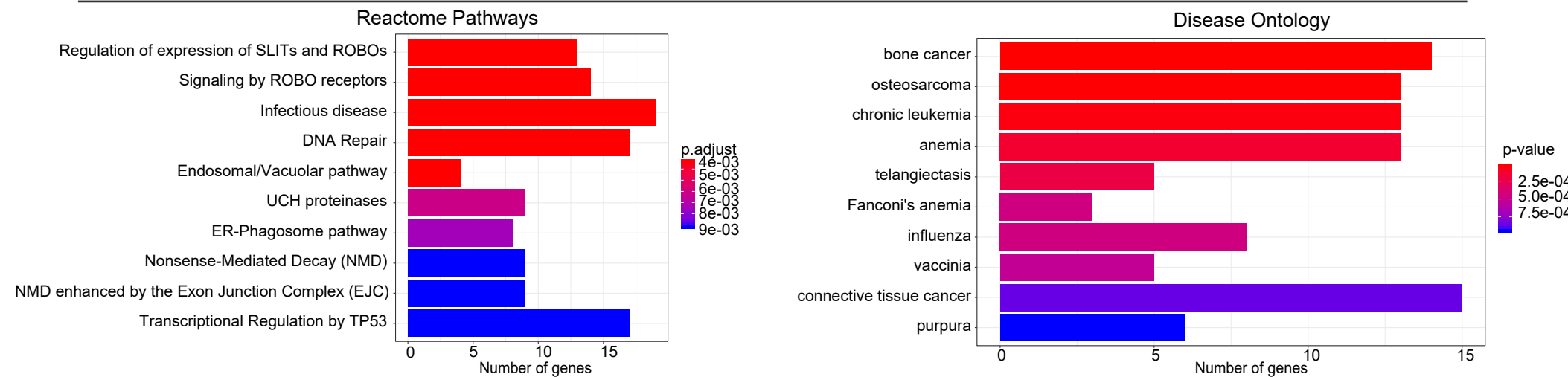

B

## HD-MY-Z, unique switching genes

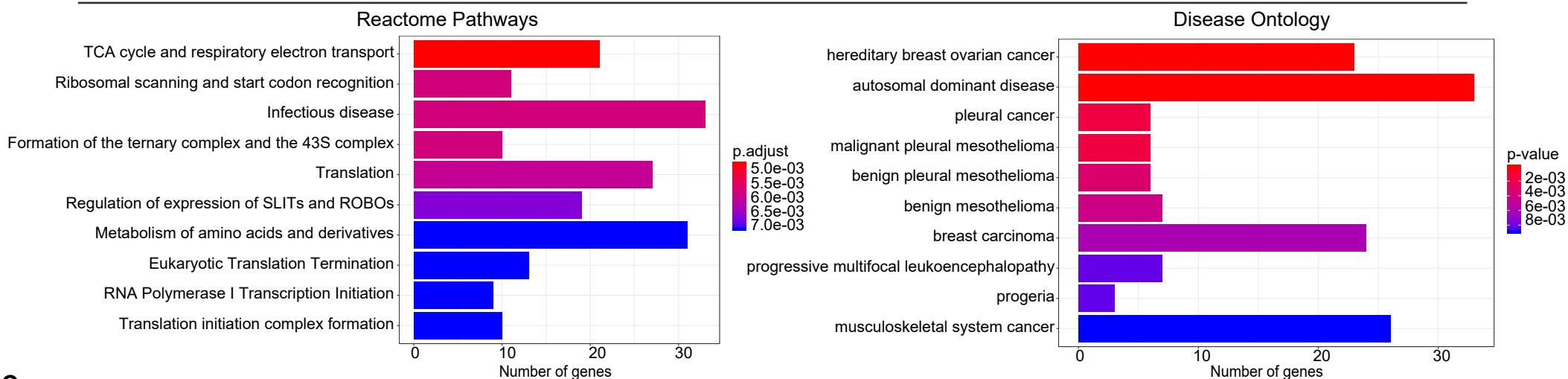

C

## L-1236, unique switching genes

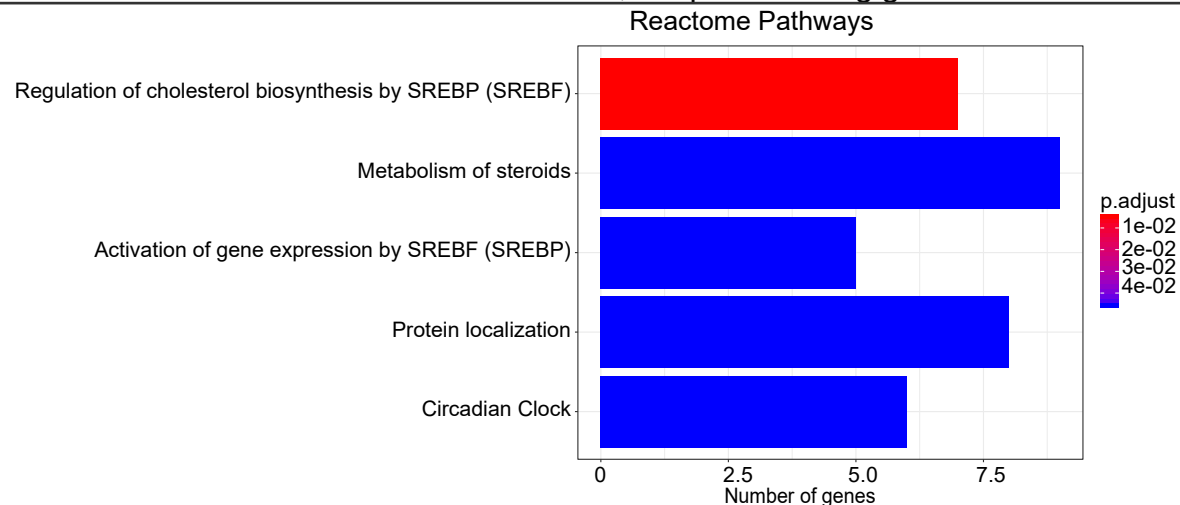

Supplement: Supplementary file 1 [file ijms-20-01182-s001.zip › Genovetal_Figure_S4_13.02.2019.pdf]

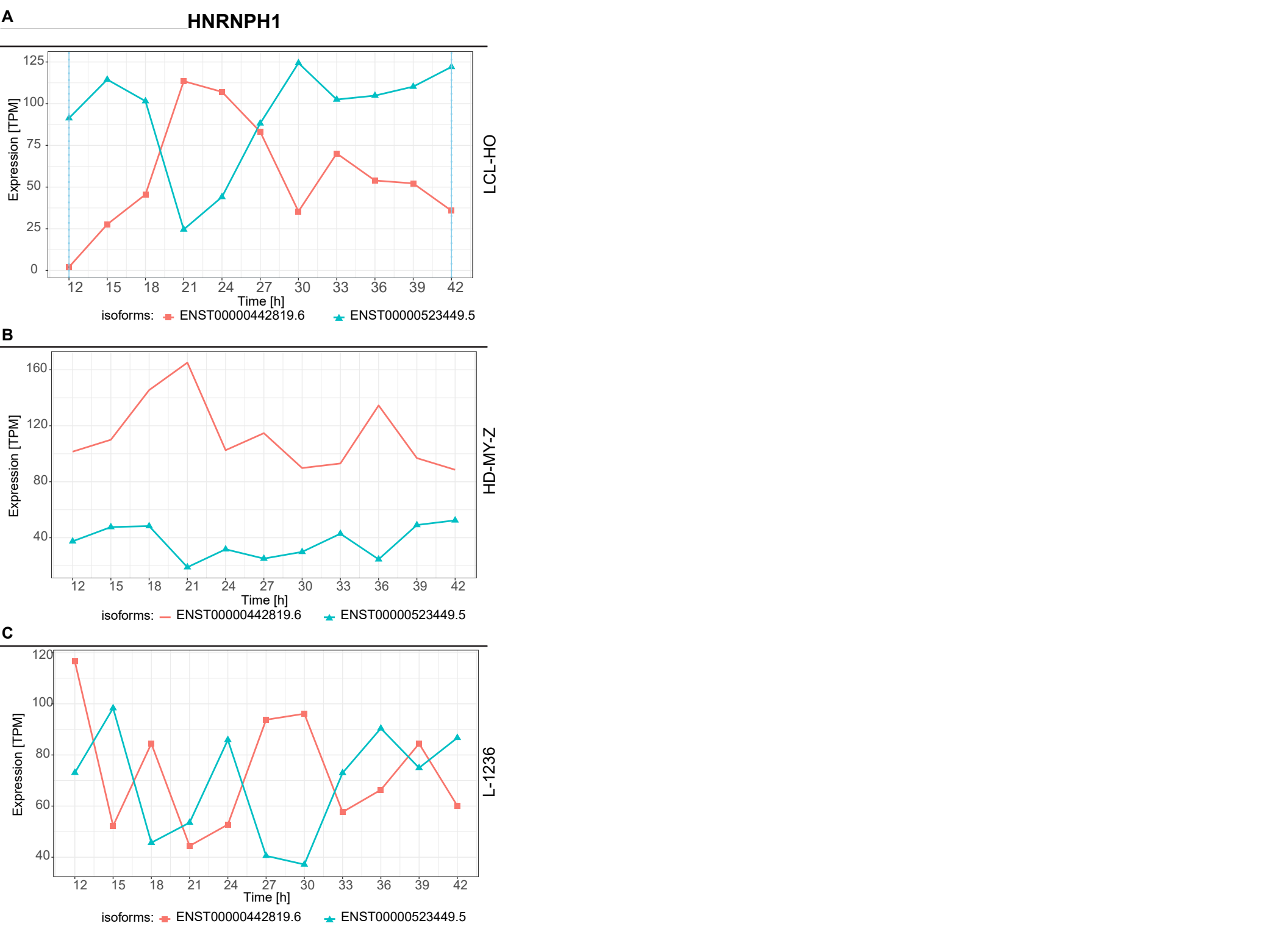

Supplement: Supplementary file 1 [file ijms-20-01182-s001.zip › Genovetal_Figure_S5_13.02.2019.pdf]
