# Supplementary material for: Temporal Splicing Switches in Elements of the TNF-Pathway Identified by Computational Analysis of Transcriptome Data for Human Cell Lines
Source: Int J Mol Sci. 2019 Mar 8;20(5):1182. doi: 10.3390/ijms20051182 (PMC6429354; doi:10.3390/ijms20051182)
Supplement: Supplementary file 1 [file ijms-20-01182-s001.zip › Genovetal_manuscript_15022019.doc]

## Temporal splicing switches in elements of the TNF-pathway identified by computational analysis of transcriptome data for human cell lines

**Short title: Splicing switches in human cell lines**

Nikolai Genov^1,2^, Alireza Basti^1,2^, Mónica Abreu^1,2^, Angela Relógio^1,2,*^

^1^Charité - Universitätsmedizin Berlin, corporate member of Freie Universität Berlin, Humboldt - Universität zu Berlin, and Berlin Institute of Health, Institute for Theoretical Biology, Germany

^2^Charité - Universitätsmedizin Berlin, corporate member of Freie Universität Berlin, Humboldt - Universität zu Berlin, and Berlin Institute of Health, Medical Department of Hematology, Oncology, and Tumor Immunology, Molecular Cancer Research Center, Germany

^*^Corresponding author: angela.relogio@charite.de

## ABSTRACT

Alternative splicing regulates key cellular processes and its dysregulation is associated to several pathologies including cancer. Although some studies point to a regulation of alternative splicing and its effector mechanisms in a time-dependent manner, the extent and consequences of such a regulation remains poorly understood. In the present work, we investigated the time-dependent production of isoforms in two Hodgkin Lymphoma cell lines of different progression stages (HD-MY-Z, stage IIIb and L-1236, stage IV) compared to a B lymphoblastoid cell line (LCL-HO) with a focus on TNF pathway-related elements. For this, we used newly generated time-course RNA-sequencing data from the mentioned cell lines and applied a computational pipeline to identify genes with isoform-switching behaviour in time. We analysed the temporal profiles of the identified events and evaluated in detail the potential functional implication of changes in isoform expression for the selected top-switching genes. Our data indicate that elements within the TNF pathway undergo a time-dependent variation in isoform production with putative impact on cell migration, proliferation and apoptosis. These include the genes *TRAF1*, *TNFRSF12A* and *NFKB2*. Our results point to a role of temporal alternative splicing in isoform production, which may alter the outcome of the TNF pathway and impact on tumorigenesis.

**Key words:** circadian clock; cancer; systems biology; high-throughput data analysis; alternative splicing, analysis of oscillating genes, pathway enrichment, RNA-sequencing, TNF-pathway

## INTRODUCTION

Alterative splicing (AS) belongs to one of the key biological processes, which regulates gene expression diversity and function in eukaryotes. In this process, different coding regions of the pre-mRNA are included in the mature mRNA in a combinatorial way. This leads to exon/intron inclusion or exclusion events in the final transcript form of a gene. As a result, AS is able to generate a myriad of functional proteins from the same gene. This impacts on cellular functions and pathways, and provides the organism an evolutionary advantage to adapt to its changing environment. Remarkably, it was estimated that around 95% of human genes are alternatively spliced . Hence, AS can influence different aspects of protein functionality including protein localization, protein-protein binding and catalytic properties of proteins . Also cellular phenotypes (e.g. apoptosis and proliferation), as well as regulation of gene expression and histone modification can be fine-tuned by AS events . Therefore, failures in splicing decisions (e.g. due to genetic mutations leading to sequence alterations within the transcript, which may alter the sequences of splice sites) or within the splicing machinery (e.g. in small nuclear ribonucleic particles snRNPs) may lead to the generation of aberrant transcripts and contribute to human disease .

In cancer, changes of AS are well studied, and a deregulated AS is reported as one of the hallmarks of cancer . Several protein isoforms generated by alternative splicing are crucial for cancer progression and are the subject of experimental therapeutic interventions . One example is the RON (tyrosine kinase receptor) gene, which is involved in cell motility and invasion. A constitutively active isoform of this gene (delta-Ron) is generated via the skipping of an alternative exon regulated by the splicing factors SF2/ASF. It was shown that overexpression of SF2/ASF isoform translates into the constitutively active form of the RON kinase that determines the epithelial to mesenchymal transition facilitating invasion, intravasation and extravasation, known steps of metastasis . Another example is the splicing factor SRSF6, which via a fine-tuning of its expression, is able to alter the splicing of several oncogenes, including the insulin receptor (INSR), the kinase Mnk2 (MKNK2) and the discs large MAGUK scaffold protein 1 (DLG1) leading to the production of oncogenic isoforms or to the reduction of the tumour-supressing isoforms .

Several studies have illustrated a cancer-specific pattern of AS in various cancer types (e.g. in neuroblastoma, Hodgkin lymphoma, prostate, colon and lung cancers) . Particularly, the analysis of AS using microarray data in Hodgkin lymphoma (HL) revealed stage–specific patterns of AS between HL cell lines of different tumour stages . Interestingly, HL tumours of the same stage (HDML-2 and L-540, stage IV) showed greater similarity in their splicing pattern (70% similarity) when compared to a HL cell line from a different stage (HD-MY-Z, stage IIIb, 55% similarity). These patterns also enabled the establishment of a differential genetic signature between HL tumours and a non-tumour B lymphoblastoid cell line (LCL-HO). Additionally, several splicing factors (e.g. hnRNP E1, U2AF65 and NOVA-1/2) were reported has differentially expressed between HL cell lines and the B lymphoblastoid cell line LCL-HO, highlighting the prevailing function of splicing in tumorigenesis in Hodgkin lymphoma cells .

Increasing evidence points to the regulation of several post-transcriptional processes in a time-dependant manner. Genes and proteins hallmarked by circadian (ca. 24h period) and ultradian (between 8h – 12h period) expression were shown to be conserved across several species and regulate several pathways . These include AS, polyadenylation, mRNA stability and mRNA transport . Additionally, several RNA-binding proteins involved in AS regulate the expression, localization and translation of core-clock components (e.g. *Per1* and *CLOCK*) . Altogether, these findings indicate a role of splicing in the regulation or fine-tuning of the circadian machinery and vice versa with a potential impact in tumorigenesis.

One of the pathways for which published data points to a role of AS in the regulation of key players of the signalling cascade is the TNF pathway . As demonstrated by many studies, TNF plays a central role in diverse cellular events such as inflammation, immunity, cell proliferation, differentiation and apoptosis . Hence, TNF can activate several downstream elements to induce differential cellular responses through the activation of TNF receptor elements (TNFRs), members of the TNF superfamily receptors. This activation, in turn, can induce the initiation of diverse signalling cascades leading to apoptosis (via the TNFR1-associated death domain protein TRADD) or inflammation and proliferation (via the activation of TNF receptor-associated factors TRAFs, NF-kB activation as well as the c-Jun N-terminal kinase (JNK)-dependant kinase cascade) . As a consequence, TNF is involved in many aspects of tumorigenesis by inducing tumour suppression (activating apoptosis as described above) of tumour initiation and progression mostly via the activation of NF-kB- and activator protein 1 (AP-1)-dependant pathways , or enhancing angiogenesis through various angiogenic factors such as the vascular endothelial growth factor (VEGF) and the JNK-dependant pathway . Given the above described AS properties, it is conceivable that AS-generated isoforms within the elements of the TNF signalling pathway may play a major role in the balance between TNF-induced survival, inflammation and apoptosis.

Despite the increasing relevance of the time-dependant regulation of cellular processes in cancer progression, the impact of circadian regulation in splicing remains poorly understood. Thus, it is of great biological and clinical relevance to identify timely regulated AS events on elements of the TNF pathway in cancer cellular models. Yet, additional studies at the transcriptome level using circadian data sets are still rare and are essential for the investigation of time-dependent AS regulation .

In this work, we characterized the extent of a time-dependent isoform generation in elements within the tumour necrosis factor (TNF) pathway. We generated 33 time-course data sets (sampling every three hours between ZT12h and ZT42h) to investigate the time-dependant production of isoforms in two HL cell lines at different stages (HD-MY-Z, stage IIIb and L-1236, stage IV) compared to a B lymphoblastoid cell line (LCL-HO) with a focus on TNF pathway-related elements. Using a computational approach, we identified several genes involved in the TNF pathway to undergo a time-dependent change in isoform expression with potential implications on the output of the pathway. These include TNF receptor-associated factor 1 (TRAF1), TNF receptor superfamily 12A (TNFRSF12A) and the nuclear factor kappa B subunit 2 (NFKB2) with relevant functions in cell proliferation, migration and apoptosis. Our results further emphasize the potential impact of AS in cellular physiology, in a cancer context, and highlight the effect of temporal isoform switching events as mediators of these processes.

## RESULTS

**3.1 Computational analysis of RNA-seq time series data reveals a high number of time-dependent splicing switches between isoforms in the LCL-HO, HD-MY-Z and L-1236 cell lines**

Previous research pointed at the relevance of splicing in cancer, and identified cell-specific AS changes in Hodgkin Lymphoma cell lines . In order to investigate the time-distribution of the isoforms detected in haematological cell lines we used a B lymphoblastoid cell line LCL-HO, the Hodgkin lymphoma cell lines HD-MY-Z (stage IIIb) and L-1236 (stage IV) and generated a total of 33 time-course data sets (sampling every three hours between ZT12h and ZT42h), which were subsequently sequenced. We used the 33 data sets as the input of our computational analysis pipeline (**Figure 1A**). We quantified paired-end RNA-Seq data with Salmon by using the EMBL reference transcriptome Homo_sapiens.GRCh38.cdna. We used the quantified transcripts in TPM (transcripts per million) to analyse the isoform expression profiles over time, and identified switches in the expression of isoforms from the same set of genes with the TSIS R package , and further investigated the switches regarding potential functionality. Isoform switching is defined as the reversal of relative abundance of different isoforms of the same gene over time. Interestingly, the number of switches and their distribution over time varies greatly between the cell lines (**Figure 1B**). We filtered the switching events with a p-value cut-off of 0.001, and a difference cut-off of 1 as suggested in the TSIS package (see Materials and Methods for details) . The difference cut-off is defined as the sum of average abundance differences in TPM of the two isoforms in the intervals before and after the switch. This indicates the magnitude of the switch. Higher values mean larger changes in expression before and after the switch. Once we identified potential switches, we performed downstream analysis with the IsoformSwitchAnalyzeR R package for coding potential and signatures of functional domains. Furthermore, we performed a Reactome pathway enrichment analysis for the groups of genes containing isoform switches. For the LCL-HO we detected 881 switches in pairwise isoform expression in time (**Table S1**) with one switch time-peak (STP) containing 273 switching events in pairwise isoform expression in a 3h bin between 27h and 30h. A STP is defined as a peak in the distribution of isoform switching events per 3h bin as computed by the TSIS algorithm. For the HD-MY-Z we detected 1865 switches between isoforms (**Table S2**) and one STP with 783 switching events in pairwise isoform expression in a 3h bin between 33h and 36h. For the L-1236 cell line we detected 755 switches between isoforms (**Table S3**) and three STPs at 18h to 21h (166 switching events), 24h to 27h (167 switching events) and 36h to 39h (148 switching events), as depicted in **Figure 1B**. Interestingly, this might indicate a specific pattern of STP distribution that could correlate to the clinical stage of the cancer (HD-MY-Z vs L-1236, so that the higher stage cell line contains more STPs.

Figure 1: Analysis of isoform expression switching over time shows an enrichment of switching events in a time-specific manner (A) Schematic representation of the pipeline for the detection and analysis of isoform switching events. (B) Distribution of isoform switching events for all genes over time. The LCL-HO cell line shows only one switch time-peak (STP) between 27h and 30h (273 switching events), the HD-MY-Z cell line shows a STP between 33h and 36h and the L-1236 cell line shows three STPs between 18h to 21h (166 switching events ), 24h to 27h (167 switching events) and 36h to 39h (148 switching events)

**3.2 The STPs are characterized by trends in functional changes such as the transcript coding potential, domain contents and sensitivity to nonsense-mediated decay**

We further investigated the switches between isoforms in time, focusing in the region around the STPs, and tested for potential functional properties of the resulting transcripts on the sequence level (**Figure 2, Figure S1-S3**). Such properties include the coding potential of the transcripts (CP), the potential protein domains identified based on the sequence of the transcript (Domains identified), intron retention properties (IR), the susceptibility of the transcript to nonsense-mediated decay (NMD), and the similarity of the ORF sequence between the time-points (ORF seq similarity). Remarkably, we found large differences in the functional outcome of the switching event between the cell lines LCL-HO and HD-MY-Z **(Figure 2A and B, respectively**). For the LCL-HO cell line the STP between 27h and 30h shows an increase of coding transcripts combined with longer ORFs and domain gains **(Figure 2A)**, however this difference is not statistically significant (**Figure S1**). For the STP of the HD-MY-Z cell line between 33h and 36h we detected a significant increase **(Figure S1)** of the number of non-coding transcripts and therefore domain loss in the sequence with corresponding shorter ORFs **(Figure 2B)**. The three STPs of the L-1236 cell line lead to interesting observations **(Figure 2C-E)**. The first STP between 18h and 21h shows a statistically significant (with confidence interval of 95%) increase of coding transcripts with domain gains and longer ORFs **(Figure 2C and Figure S1)**. The second and third STPs (between 24h to 27h and 36h to 39h, respectively) show several switches towards isoforms with shorter ORFs and domain loss **(Figure 2D and E, respectively)**. Based on our findings, the STPs between 24h to 27h and 36h to 39h in the L-1236 cell line are similar to the STP of HD-MY-Z between 33h and 36h, which could indicate a potential relevance of the temporal distribution of the STPs to its functional outcome in each cell line. The changes in transcript length and potential functionality between the STPs relate to changes in the alternative splicing events over time (**Figure S2-S3**). In all cases, alternative transcription stop and start-sites are the mechanisms by which most of the changes in isoform balance are generated.

Figure 2: Computational analysis of the isoforms involved in the STPs shows that the loss of coding functionality and potential domain loss are the main consequences of changes in isoform expression. We performed a computational analysis on the highly expressed genes and isoforms with changing expression in each STP. The analysis included Coding Potential (CP), identification of potential domains (Domains ident.), possible intron retention (IR), Nonsense Mediated Decay (NMD) and Open Reading Frame (ORF) length and availability (ORF seq similarity). (A) The STP between 27h and 30h for the LCL-HO cell line shows an increase in coding transcripts for the upregulated isoforms leading to gains in potential domains and increase of ORF length. (B) The STP between 33h and 36h for HD-MY-Z shows increase of non-coding transcripts and a related loss of potential domains. The shorter ORF or its complete loss mean that sequence elements coding for potential domains are lost. (C) The STP at 18h to 21h for the L-1236 cell line shows an increase in coding transcripts for the upregulated isoforms leading to gains in potential domains, and increase of ORF length. (D – E) The STPs at 24h to 27h and 36h to 39h (respectively) for the L-1236 cell line show an increase of non-coding transcripts and a related loss of potential domains with an increase in both complete ORF loss and shorter ORFs.

**3.3 The genes for which switches in isoform expression were detected in each cell line show a specific enrichment of functionality focused in the SLIT/ROBO, SREBP and Circadian Clock pathways**

On the gene level, the switches detected for all cell lines vary between 547 genes with switches between isoform-pairs for the LCL-HO cell line (**Figure 3A, Table S4**), 958 genes with switches for the HD-MY-Z cell line (**Table S5**) and 460 genes with switches for the L-1236 cell line (**Table S6**). Some genes have multiple pairs of isoforms switching in time hence the number of switching events is greater than the number of genes. A core set of 89 genes with isoform switches is common between the three cell lines (**Table S7**). This core set shows enrichment of Reactome pathway terms related to translation and splicing (adjusted (BH) p-value 8.360E-07) as well as NMD (adjusted (BH) p-value: 1.870E-03) (**Figure 3B, Table S8**). Additionally, we performed an enrichment analysis for disease ontology pathways using the 89 gene sets (**Figure 3C, Table S9**). The genes with switches identified by our data (e.g. *VEGFA*) are involved in a variety of cancers including skin, pancreatic and colon cancer as well as lung disease according to our enrichment analysis, which is in agreement with previous studies for the mentioned gene . However, when analysing the gene sets of the individual cell lines for which switching in isoform production was detected we observed a more diverse scenario (**Figure 3D, Table S10-S12**). For the B lymphoblastoid LCL-HO cell line (547 genes with identified switches), there is an enrichment of ROBO-related pathways (**Table S10,** adjusted (BH) p-value 7.099E-08). The SLIT/ROBO pathway have important roles in tumorigenesis, cancer progression and metastasis and thus acts as a regulator for multiple oncogenic signalling pathways including the CD20, mTOR (mammalian target of rapamycin), VEGF (vascular endothelial growth factor) and EGFR (Epidermal Growth Factor Receptors) pathways . The ROBO functionality can also be found in the enriched pathways for the HD-MY-Z cell line (**Table S14**) regulation of expression of SLITs and ROBOs, adjusted (BH) p-value 6.918E-03), interestingly only in the genes with switches that are unique to this cell line (620 unique genes with identified switches) **Figure S4**), hence different genes of the same pathway are affected. The L-1236 cell line (460 genes with identified switches), however, shows an interesting enrichment of the FGFR2 (**Table S12,** fibroblast growth factor receptor 2, adjusted (BH) p-value 3.640E-04) signalling pathway, which is associated with disrupted organogenesis leading to cancer . Additionally, we performed enrichment analysis on the set of unique genes for each cell lines for Reactome pathways and disease ontology **(Figure S4, Table S13-S15**). In addition to RNA splicing and translation-related pathways, we found metabolic (metabolism of steroids, p-value 4.542E-02), the SREBP (sterol regulatory element-binding proteins) pathway (regulation of cholesterol biosynthesis by SREBP, p-value 5.090E-03 and activation of gene expression by SREBF, p-value 4.542E-02) and the circadian clock pathway (p-value 4.542E-02) to be enriched in the L-1236 cell line **(Table S15)**. SREBP belongs to transcription factors that regulate the expression of genes required for the synthesis of fatty acids and cholesterol and thus is relevant for cellular metabolism. For the LCL-HO cell line, the disease ontology enrichment for the unique switching genes shows cancer-related terms (**Figure S4**, **Table S16**). For the HD-MY-Z cell line, this result is more striking with 7 out of the top 10 disease ontology enriched terms being cancer-related (**Table S17**).

Figure 3: A core of genes with switches between isoform expression over time is common to the cell lines analysed (A) We detected an intersection of 89 genes for the three cell lines for the genes for which switching in isoform expression was detected in our analysis. (B) The top enriched pathways for the 89 genes are mRNA splicing and translation as well as the Processing of Capped Intron-Containing Pre-mRNA. (C) All but one of the top 10 enriched disease ontology terms associated with the 89 genes are different forms of cancer. (D) We analysed all genes for which switches in isoform expression over time we detected for the three cell lines. A set of common pathways including mRNA splicing, translation and ribosomal activity was identified for all cell lines, the L-1236 cell line shows an additional enrichment for the FGFR2 pathway.

**3.4 Several genes display switching in isoform production over time**

We computed the difference in expression between the switching isoforms with the TSIS package and generated lists with all switching events for the three cell lines (**Table S1-S3**) and additional short lists containing the top 20 genes with switching events for each cell line (**Table S4-S6**). We plotted several gene isoforms out of the top 20 events (**Figure 4**) to illustrate the temporal changes in the expression of the isoforms. For the LCL-HO cell line *HNRNPH1* (heterogeneous nuclear ribonucleoprotein H1) shows an absolute difference of expression of 701.44 TPM between the isoforms ENST00000262584.7 and ENST00000526668.5. *JCHAIN* (joining chain of multimeric IgA and IgM) shows an absolute difference in expression of 107.69 TPM between the isoforms ENST00000510437.5 and ENST00000543780.5 and *NME2* (NME/NM23 nucleoside diphosphate kinase 2) shows an absolute difference in expression of 408.09 TPM between the isoforms ENST00000513177.5 and ENST00000512737.5. The *HNRNPH1* gene is involved in splicing. It is overexpressed in many cancer types (e.g. rhabdomyosarcoma, esophageal squamous cell carcinoma and breast cancer) and is required for cancer growth and development . Notably, the expression of the two isoforms of *HNRNPH1* is different over time for the three investigated cell lines (**Figure S5**). There is no switch between the expression of the same two isoforms of *HNRNPH1* in HD-MY-Z and multiple switches in the data for L-1236. JCHAIN is involved in innate and adaptive immune response, and is differentially expressed in different cancer types (e.g. colon and prostate cancer) . NME2 is involved in myeloid differentiation, apoptosis and cell development. It acts as a transcriptional activator of the *MYC* gene and its isoforms, which are either differentially or ubiquitously expressed .

For the HD-MY-Z cell line the *CD44* gene shows an absolute difference in expression between the isoforms ENST00000263398.10 and ENST00000352818.8 of 164.95 TPM. The *TNFRSF12A* gene shows an absolute difference in expression of 211.08 TPM for the isoforms ENST00000326577.8 and ENST00000341627.5 and the *HNRNPA1* (heterogeneous nuclear ribonucleoprotein A1) gene (involved in splicing and mRNA transport) shows an absolute difference in expression of 171 TPM for the isoforms ENST00000340913.10 and ENST00000547566.5. *CD44* encodes for a cell-surface glycoprotein and can act both as a growth‐ and invasiveness‐promoting molecule and as a tumour‐suppressing cofactor via its different splice variants . TNFRSF12A belongs to the TNF superfamily, it is involved in angiogenesis, cell migration and apoptosis.

For the L-1236 cell line the *NFKB2* gene shows an absolute difference of 84.39 TPM between the isoforms ENST00000189444.10 and ENST00000467116.5, the *PPP4C* (protein phosphatase 4 catalytic subunit) gene shows an absolute difference of 147.32 TPM between the isoforms ENST00000279387.11 and ENST00000561610.1. The *HSPD1* (heat shock protein family D (Hsp60) member 1) gene shows an absolute difference in expression of 348.472 TPM between the isoforms ENST00000388968.7 and ENST00000345042.6. *PPP4C* mediates the repair process of damaged DNA upon double strand break and *HSPD1* is involved in B and T cell activation, among other processes. NFKB2 is a member of NF-KB family of proteins and of TNF signalling pathway. It is involved in transcription, differentiation, apoptosis and inflammatory response. Altogether, these results indicate that a time-dependant change in isoform expression is present in genes with impacts on vital cellular processes. Interestingly, several of these genes are elements of the TNF pathway.

Figure 4: Several of the genes with largest expression difference between the switching isoforms are related to the TNF signalling pathway. (A) Out of the top 20 genes with largest expression difference between the isoforms for the LCL-HO cell line are the *JCHAIN*, *NME2* and *HNRNPH1* genes, which depict remarkable difference in expression patterns over time. (B) Central genes involved in cancer metastasis such as *CD44* undergo switching in isoform expression in HD-MY-Z. *TNFRSF12A* is related to the TNF signalling pathway. (C) The changes in the expression of NFKB2 in L-1236 are associated to the TNF signalling pathway, and suggest possible rhythmicity of isoform expression switching. Also the expression changes between isoforms for *PPP4C* show a circadian pattern.

**3.5 Multiple genes involved in the TNF pathway or related to TNF signalling switch between isoforms in a time-dependent manner**

Interestingly, several of the top switching genes such as *TNFRSF12A* and *NFKB2* are related to the TNF pathway. Therefore, we investigated the TNF pathway in detail and detected multiple switches in isoform expression over time in genes such as *TRAF1* and *RACK1* (receptor for activated C kinase 1) in the LCL-HO cell line **(Figure 5A)**, *RACK1* and *SHARPIN* (SHANK associated RH domain interactor) in the HD-MY-Z cell line **(Figure 5B)**, as well as in *UBB* (ubiquitin B) and *RPS27A* (ribosomal protein S27a) for the L-1236 cell line (**Figure 5C**).

Figure 5: Multiple genes from the TNF pathway show switching between the isoform expression over time (A) For the LCL-HO cell line the *TRAF1* and *RACK1* genes show a differential expression of the corresponding isoforms. (B) For the HD-MY-Z cell line the *RACK1* gene shows switching behaviour between the isoforms. Multiple isoforms of the gene show a switch phenotype over time. The *SHARPIN* gene shows switching of isoform expression at 36h-39h, corresponding to STP1 for the cell line HD-MY-Z. (C) For the L-1236 only the genes *UBB* and *RPS27A* are affected by switching in time between isoform expression within the TNF pathway.

The presence of switches in isoform expression over time and their concentration at specific time points has potential functional implications on the function of the generated protein and the subsequent alterations in the phenotype of the cell. To investigate this aspect in more detail, we performed an analysis on the sequence level with the R package IsoformSwitchAnalyzeR that allows for the integration of data from tools such as CPAT (Coding-Potential Assessment Tool) for coding potential analysis and PFAM (protein family) for potential protein domain identification. We focused on the genes *TNFRSF12A, CD44* and *NFKB2* in more detail given their role in the multiple cellular pathways and cancer and the high scoring of the corresponding switching events. We investigated all isoforms with high expression for the corresponding genes. We observed large changes in expression for the *TNFRSF12A* gene in the HD-MY-Z cell line (**Figure 6A**). For *TNFRSF12A* the switch between ENST00000326577.8 and ENST00000341627.5 isoforms leads to a change between a coding and a non-coding isoform, meaning that the corresponding stn_TNFRSF12A domain will be affected and the non-coding isoform contributes more to the summed up expression of the gene. Similarly, while multiple isoforms of *CD44* are affected by changes over time, this effect is most dramatic regarding the non-coding isoform ENST00000528086.5, with a signal that nearly disappears within 9 hours **(Figure 6B)**. Remarkably, the overall cumulative expression of the gene shows only a minor change of 4.05% from 773.27 TPM to 804.55 TPM, underscoring the importance of isoform-level investigation. Similarly, the genes *NME2* and *JCHAIN* in LCL-HO show larger changes between the expression of isoforms than the total expression of the gene (**Figure S6**). For the L-1236 cell line, we identified a switch with potential major implication between the isoforms ENST00000189444.10 and ENST00000467116.5 of *NFKB2*. ENST00000467116.5 does not contain the important death domain, central in the regulation of apoptosis and inflammation, additionally the multiple Ank (Ankyrin) domains are absent. These are critical for folding and stability and are involved in protein-protein interactions. As mentioned above, the cumulative expression of the gene shows little variations (6.93% from 411.96 TPM to 382.52 TPM) whereas the expression of the isoforms shows strong differences within six hours. These findings point to the potential implication of temporal switching events in isoform expression and its impact in the subsequent protein functions.

Figure 6: Genes related to the TNF signalling pathway show switching between isoform expression with potential functional implications (A) Based on the analysis of switching behaviour between isoforms (Figure 4A), we further investigated the potential functionality of the isoforms. The two isoforms of *TNFRSF12A* have different predicted functionality. ENST00000326577.8 has predicted coding functionality and a stn_TNFRSF12A domain, whereas the ENST00000341627.5 is predicted to be non-coding. (B) The *CD44* gene with its multiple isoforms illustrates the importance of alternative splicing: the total gene expression is almost unchanged between the time points 30h and 39h, however the individual isoforms show a larger deviation in expression. Most notably, the ENST00000425428.6 isoform, predicted to be non-coding, is almost absent at 39h. (C) In the L-1236 cell line we detected changes in isoform balance in the *NFKB2* gene (Figure 4C). The total expression of the gene shows only minor change between the time points, however the isoform ENST00000467116.5 lacking the important death-domain is upregulated at 30h as compared to 24h (STP2).

The abundance of time-dependant changes of isoform expression in the TNF pathway (**Figure 7**) points to a possible change of the output of the pathway within a day.

The TNF pathway is activated through the binding of TNF to its receptors (TNFR1 and TNFR2) leading to the activation of several signalling cascades. While the activation of the TNFR1 mainly results in the activation of cellular components leading to cell apoptosis, TNFR2 induction is required for cell survival and inflammation responses . One of the main components of the latter pathway is the TRAF complex (TRAF1, TRAF2 and inhibitor of apoptosis proteins cIAPs), which regulates several downstream signalling pathways, including survival and inflammation through the NF-kB pathway and cell apoptosis via interactions with the LUBAC (linear ubiquitin assembly complex) .

Results from our computational analysis revealed that in all of the investigated cell lines, we found a TNF or TNF-related element with temporal changes in isoform production. We identified *TRAF1* in the LCL-HO cell line, *TNFRSF12A* and *RACK1* in the HD-MY-Z cell line and *NFKB2* in the L-1236 cell line, which show significant isoform switching behaviour and can regulate the resulting output of the TNF signalling pathway in this cellular model system.

Figure 7 Elements within the TNF pathway possess a time-dependent switching behaviour in isoform expression: TNF can activate different pathways to induce cell survival, inflammation or apoptosis. Cell apoptosis is induced by the activation of Caspase 8 through TRADD and FADD. TRAF1/TRAF2 complex mediates cell survival and inflammation via the JNK-dependent cascade, MEKK cascade and the NF-kB activation by RIP. Elements, which display time-dependant switches in isoform expression are marked in colour with their associated genes marked red. Yellow: elements identified in the HD-MY-Z cell line. Green: elements identified in the LCL-HO cell line. Purple: elements identified in the L-1236 cell line.

## DISCUSSION

In the present study, we characterized the existence of time-dependent AS events in HL cell lines (HD-MY-Z, stage IIIb and L-1236, stage IV) and a non-tumour B lymphoblastoid cell line (LCL-HO) using a computational approach. Stage-specific patterns of AS and splicing factor expression for HL cells from different tumour stages has previously been reported . The authors made use of these patterns to classify the stage of each tumour and to distinguish between a non-tumour B lymphoblastoid cell line and cancer cells . To date no correlation between the splicing events and the circadian clock have been investigated for this cellular system.

***Differential number of splicing switches in cancer cell lines correlate with tumour progression stage***

Published data points to the regulation of AS, and various post-translational processes by the circadian clock pointing to a time-dependency of such molecular mechanisms with implications in cellular function . To investigate this in detail, we performed a time-course genome-wide screening of the two HL cell lines and the non-tumour B lymphoblastoid cell line and carried out a detailed computational analysis of the generated 33 sets of RNA-seq data. We identified cell-specific isoform switching events, predicted the functional outcome of the identified switches. Our data indicates a distinct and unique pattern of switching behaviour in the investigated cell lines, which could possibly correlate with their specific tumour stage. Comparing the two Hodgkin lymphoma cell lines in our study, we identified a higher number of STPs (three) available in the higher stage cancer cell line (L-1236, stage IV) than the lower stage cell line (HD-MY-Z, stage IIIb) with only one STP (**Figure 1B**). Moreover, while the changes in potential consequences (such as ORF length and domains present) in the STP of LCL-HO are present, they are not statistically significant. In contrast, in HD-MY-Z, which displays one STP, the changes in potential functional consequences of pairwise expression switching are significant. Also for L-1236, the two STPs have significant changes and even opposing changes in potential consequences of the pairwise expression switching of isoforms. Through enrichment analysis for the genes that undergo temporal changes in isoform expression in each cell line, we identified several pathways, which are related to cancer (SLIT/ROBO and FGFR2 signalling pathway). Previous studies indicate that the SLIT/ROBO pathway acts as master regulator for multiple oncogenic signalling pathways including the CD20, mTOR (mammalian target of rapamycin), VEGF (vascular endothelial growth factor) and EGFR (Epidermal Growth Factor Receptors) pathways in several cancer types including lung cancer, colon cancer and lymphoma . Similar to the SLIT/ROBO pathway, the FGFR2 signalling pathway plays roles in cancer progression as well. Since FGFR2 pathway interacts with most of the other major signalling pathways, including BMP, WNT, Notch and Hedgehog, its aberrant activity is associated with developmental defects resulting in metabolic disorders, and cancer including lymphoma (reviewed in ).

We further analysed a reduced set of genes that present splicing switches in a time-dependent manner. In particular, out of the top 20 genes with largest expression difference between the isoforms we identified the *JCHAIN*, *NME2* and *HNRNPH1* genes (for the LCL-HO cell line), *CD44*, *TNFRSF12A* and *HNRNPA1* (for the HD-MY-Z cell line) and *NFKB2*, *PPP4C* and *HSPD1* (for the L-1236 cell line) which play important roles in tumorigenesis. Transcript variants of HNRNPH1 were shown to be differentially involved in tumorigenesis. Overall, positive expression of HNRNPH1 is associated with poor tumour differentiation degree . The NME2 gene can act as a metastasis suppressor by altering telomere function and telomere length in lung cancer and fibroblasts. . CD44 is epigenetically regulated in Hodgkin and non-Hodgkin lymphomas and is reported to be unmethylated in the L-1236 cell line . Several studies have identified various isoforms of the CD44 gene . Particularly, the CD44v isoforms are involved in specific signalling pathways. While the CD44v3 isoform bind several heparan sulfate‐binding growth factors such as fibroblast growth factors (FGFs) and heparin‐binding epidermal growth factor (EGF), the CD44v6 isoform contains a binding site for hepatocyte growth factor (HGF) and vascular endothelial growth factor (VEGF) inducing CD44v6/HGF/cMet complex formation, leading to c‐Met or HGF‐induced Ras signalling activation . Therefore, cancer cells often express a variety of CD44 isoforms to regulate multiple oncogenic pathways required for cancer progression and tumour development .

The PPP4C gene is required for the correct DNA damage repair functioning upon a double strand break. Moreover, it was shown to be involved in the TNF and NF-kB pathways. It acts as a signalling component connecting the TNF signal to the JNK pathway . HSPD1 encodes a heat-shock family protein chaperone that exhibits elevated expression in Hodgkin’s and large cell lymphoma and is involved in the B and T cell immune response . Altogether, these genes encode for functionally different isoforms involved in vital cellular and tumorigenic processes and hence, the temporal change in their isoform balance could imply subsequent changes in tumour progression.

***Putative impact of splicing switches in the fine-tuning of the TNF pathway: survival vs apoptosis***

It was recently reported, that splicing isoforms of the main elements within the TNF pathway can have an impact on both inflammation and cancer and hence, affect the outcome of the pathway .

Among the genes identified by our bioinformatics approach, are several members of the TNF and TNF-related signalling pathway that show a rhythmic pattern of isoform expression: *TNFRSF12A and SHARPIN* in the HD-MY-Z cell line, *NFKB2* in the L-1236 cell line and *TRAF1* in the LCL-HO cell line*.* The TNF receptor superfamily 12A gene (*TNFRSF12A*) displays a time-dependant isoform generation in the HD-MY-Z cell line. Surprisingly, one of the transcripts for which a temporal isoform generation was detected undergoes NMD, which may influence its functional outcome in time. In cancer, TNFRSF12A can promote angiogenesis and endothelial cell proliferation. It was reported that *TNFRSF12A* knockout inhibits hepatocellular carcinoma cell proliferation and migration in vitro . TRAF1 belongs to the TNF receptor-associated factors that regulates the activation of NF-kB and JNK pathways and is involved in both cell survival and apoptosis . Another important component of the TNF signalling pathway is SHARPIN, which belongs to the linear ubiquitin chain assembly complex (LUBAC) involved in the NF-kB and apoptotic signalling pathways. It was shown that SHARPIN deficiency leads to rapid cell death upon TNF stimulation via FADD- and Caspase-8-dependent pathways . Another example of a TNF-related component found in our analysis is the nuclear factor kappa B subunit 2 (NFKB2) for which we identified splicing switches in the L-1236 cell line. Studies have indicated that NFKB2 may be involved in HL development (in L-1236) and the NF-κB-mediated transcriptional repression of the clock feedback limb could cause circadian disruption in response to inflammation in mice . In the LCL-HO cell line, TNF receptor associated factor 1 (*TRAF1*) showed a temporal pattern of isoform expression. Interestingly, yeast two-hybrid interaction assays revealed that TNFRSF12A binds to members of the TRAF family including TRAF1 and thereby activates the NF-κB signalling pathway [35]. The NF-κB signalling pathway is affected by AS events within its components, which could either inhibit or enhance the outcome response .

Taken together, the results of our study further reinforce the hypothesis, that AS could potentially fine-tune the outcome of the TNF signalling pathway in a time-dependent manner. This adds a hidden layer of temporal regulation in the TNF pathway that will require further experimental studies to fully validate and further evaluate the impact of our findings in tumorigenesis.

## MATERIALS AND METHODS

***Cell culture***

The Hodgkin lymphoma cell lines, HD-MY-Z (DSMZ – #ACC 346) and L-1236 (DSMZ - #ACC 530), and the B lymphoblastoid cell line, LCL-HO (DSMZ – #ACC 185) were maintained in RPMI 1640 (Gibco) supplemented with 1% penicillin-streptomycin (Gibco) and 10% foetal bovine serum (Gibco) and incubated at 37°C with 5% CO_2_ atmosphere.

***Sampling and RNA extraction***

Cells were seeded in 35 mm dishes with a density of 5×10^5^ cells/dish and synchronized by fresh medium addition. Cells were collected every three hours, in triplicates and the pellets were stored at -80°C. Total RNA was isolated using the RNeasy extraction kit (Qiagen), including DNase digestion, according to manufacturer’s instructions. Cells were lysed with 350 µl RLT buffer and the lysate was homogenized. Total RNA concentration was determined using NanoDrop® ND-1000 UV-Vis Spectrophotometer and stored at −80°C. A total of 33 time-course data sets (sampling every three hours between ZT12h and ZT42h) was produced for subsequent sequencing.

***RNA-Sequencing***

For RNA-sequencing the total RNA was processed by Illumina standard protocols to prepare the mRNA-Seq library, followed by deep sequencing in a bi-directional, 75-base mode on an Illumina NextSeq sequencer. The RNA-sequencing was performed in Genomics Core Facility at the European Molecular Biology Laboratory (EMBL) in Heidelberg.

***Enrichment analysis***

Enrichment analysis was performed with the clusterProfiler (ver. 3.10.0) and ReactomePA (ver. 1.26.0) R packages using Reactome Pathways. All reactome pathway enrichment was performed with a p-value cut-off of 0.05 and Bonferroni Hochberg correction for multiple testing against default “universe” background set of genes with a q-value cutoff of 0.2. Disease ontology enrichment was performed with the disease ontology as ontology of choice and p-value cut-off of 0.05 as well as q-value cut-off of 0.5.

***RNA-seq data processing***

The time course paired-end RNA-seq samples were quantified with the Salmon software package against the reference transcriptome provided by the EMBL. The transcriptome version used was Homo_sapiens.GRCh38.cdna.A single sample represents each time point, no replicates were used. Noise was added to the data to simulate potential biological variation with the R function jitter and “factor” parameter set to 10 as well as amount parameter set to 2.

***Detection and visualization of splicing switches***

For the detection of splicing switches over time the TSIS R package (ver. 0.2.0) was used with “mean” as the detection method and a spline degree of 4 to focus on isoforms with a period close to 24h. Isoform switches were visualized with the TSIS package and in detail with the IsoformSwitchAnalyzeR R package. For the TSIS package, the MEAN scoring method was used. Filtering of the results was done with TSIS with a probability cut-off of 0.5, difference cut-off of 1, p-value cut-off of 0.001, a minimal time in interval of 2, correlation cut-off of 0, lower time of 0h and upper time of 30h (experimental time) without a reduction to the most abundant isoforms. For the analysis of coding functionality, we used a coding potential cut-off of 0.725. For the visualization of pairwise comparisons between the STPs we used a difference cut-off of 0.1. Genes with single isoform are removed from the pairwise STP analysis.

Computation of abundance differences (from Guo et al. ): Metric 2 indicates the magnitude of the switch. Higher values mean larger changes in abundances before and after the switch. S2 is the sum of average abundance differences of the two isoforms in both intervals.  *S_2_(iso_i_, iso_j_|I_1_, I_2_) = d(iso_i_, iso_j_|I_1_) + d(iso_i_, iso_j_|I_2_)*

Where *d(iso_i_,iso_j_|I_k_)* is the average difference of abundances between *iso_i_* and *iso_j_* in the interval *I_k_, k=1,2* defined as

*d(iso_i_, iso_j_|I_k_) = (1/|I_k_|)∑_mIk_|exp(iso_i_|s_mIk_, I_k_) – exp (iso_j_|s_mIk_, I_k_)|*

*|I_k_|* is the number of samples in interval *I_k_* and *exp(isoi∣s_mIk_,I_k_)* is the expression of *iso_i_* of sample *s_mIk_* in interval *I_k_*.

## DATA ACCESS

The RNA-sequencing data was submitted to the GEO repository and will be release upon publication of the manuscript.

## ACKNOWLEDGMENTS

We thank Dr. Vladimir Benes and the EMBL GeneCore facility for technical support regarding the sequencing work. Work in AR’s laboratory is supported by the German Federal Ministry of Education and Research (BMBF)—eBio-CIRSPLICE - FKZ031A316 and by the Dr. Rolf M. Schwiete Stiftung. NG and AB were additionally funded by the Berlin School of Integrative Oncology (BSIO) of the Charité – Universitätsmedizin Berlin.

**AUTHOR CONTRIBUTIONS:**

Conceived and designed the work: AR; performed the wet lab experiments: MA; performed the bioinformatics analysis: NG; analysed the data: NG AB AR; contributed reagents/materials/analysis tools: AR; Wrote the paper: NG AB AR; critically read and commented on the paper: NG AB MA AR

## DISCLOSURE DECLARATION

The authors declare that there are no conflicts of interest.

**Supplementary tables legend:**

**Sup. Table S1: Pairwise switching transcripts (isoforms) after filtering and corresponding genes for the LCL-HO cell line**

**Sup. Table S2: Pairwise switching transcripts (isoforms) after filtering and corresponding genes for the HD-MY-Z cell line**

**Sup. Table S3: Pairwise switching transcripts (isoforms) after filtering and corresponding genes for the L-1236 cell line**

**Sup. Table S4: List of genes for which pairwise isoform expression switching over time was detected for the LCL-HO cell line**

**Sup. Table S5: List of genes for which pairwise isoform expression switching over time was detected for the HD-MY-Z cell line**

**Sup. Table S6: List of genes for which pairwise isoform expression switching over time was detected for the L-1236 cell line**

**Sup. Table S7: List of the 89 intersecting genes for which pairwise switching in isoform expression was detected for all three cell lines**

**Sup. Table S8: Top 10 enriched Reactome pathways for the 89 intersecting genes (Figure 3)**

**Sup. Table S9: Top 10 enriched DO terms for the 89 intersecting genes (Figure 3)**

**Sup. Table S10: Top 10 enriched Reactome pathways for all genes with switches for the LCL-HO cell line (Figure 3)**

**Sup. Table S11: Top 10 enriched Reactome pathways for all genes with switches for the HD-MY-Z cell line (Figure 3)**

**Sup. Table S12: Top 10 enriched Reactome pathways for all genes with switches for the L-1236 cell line (Figure 3)**

**Sup. Table S13: Top 10 enriched Reactome pathways for the unique genes with switches for the LCL-HO cell line (Figure S4)**

**Sup. Table S14: Top 10 enriched Reactome pathways for unique genes with switches for the HD-MY-Z cell line (Figure S4)**

**Sup. Table S15: Top 10 enriched Reactome pathways for unique genes with switches for the L-1236 cell line (Figure S4)**

**Sup. Table S16: Top 10 enriched DO terms for unique genes with switches for the LCL-HO cell line (Figure S4)**

**Sup. Table S17: Top 10 enriched DO terms for unique genes with switches for the HD-MY-Z cell line (Figure S4)**

**Supplementary figures legend:**

**Sup. Figure 1: Statistical significance testing of the functional events for the LCL-HO, HD-MY-Z and L-1236 cell lines shows multiple cases of important domain loss or gain enrichment.** The events detected in HD-MY-Z STP1 and L-1236 STP1 and STP2 contain changes with potential consequence such as domain gain/loss with a 95% confidence interval. Interestingly, L-1236 STP1 and STP2 show opposed changes.

**Sup. Figure 2:** **Switches between isoform expression in the LCL-HO and HD-MY-Z cell lines reveal alternative splicing start and termination sites as the main event present in the switches.** Testing for alternative splicing mechanisms such as Exon Skipping (ES), Multiple Exon Skipping (MES), Multiple Exclusive Exons (MEE), Intron Retention (IR), Alternative 5’ Splice Site (A5), Alternative 3’ Splice Site (A3), Alternative Transcription Start Site (ATSS) and Alternative Transcription Termination Site (ATTS) for the LCL-HO and HD-MY-Z cell lines.

**Sup. Figure 3:** **Switches between isoform expression in the L-1236 cell line reveal alternative splicing start and termination sites as the event present in the switches for all STPs.** Testing for alternative splicing mechanisms such as Exon Skipping (ES), Multiple Exon Skipping (MES), Multiple Exclusive Exons (MEE), Intron Retention (IR), Alternative 5’ Splice Site(A5), Alternative 3’ Splice Site (A3), Alternative Transcription Start Site (ATSS) and Alternative Transcription Termination Site (ATTS) for the L-1236 cell line.

**Sup. Figure 4: Enrichment of Reactome pathway and disease ontology terms points at the increasing specificity of the genes switching over time in a cell line specific manner.** (A) We performed Reactome pathway enrichment for the genes displaying switching between isoform expression in time in the LCL-HO cell line only. The ROBO pathway as well as DNA repair are enriched. Disease ontology terms related show relation to cancer. (B) We performed Reactome pathway enrichment for the genes displaying switching between isoform expression in time in the HD-MY-Z cell line. The ROBO pathway enrichment present in the LCL-HO cell line (Figure 4) is also present in the HD-MY-Z cell line. (C) Reactome pathway enrichment for the genes displaying switching between isoform expression in time in the L-1236 cell line only. A disease ontology enrichment analysis yielded no results due to the low number of genes.

**Sup. Figure 5: The development of the expression of the isoforms ENST00000442819.6 and ENST00000523449.5 of the HNRNPH1 gene over time shows marked differences between the three cell lines** (A,B,C) While switching is present in LCL-H0, in HD-MY-Z the ENST00000442819.6 isoform dominates while in L-1236 the two isoforms switch. The expression of the two isoforms is strong in all three cell lines.

**Sup. Figure 6:** **Decrease in overall gene expression for the NME2 and JCHAIN isoforms of the LCL-HO cell lines coexists with the increase of expression of individual isoforms** (A) Pairwise comparison between the time points 24h and 33h (STP) of the LCL-HO cell line for the NME2 gene. While the overall gene expression over time decreases, the expression of the ENST00000512737.5 isoform increases. (B) The decrease of expression of the ENST00000543780.5 isoform of JCHAIN over time (STP) leads to an increase in the expression of the ENST00000510437.5 isoform.

## REFERENCES

1.	Pan, Q., et al., *Deep surveying of alternative splicing complexity in the human transcriptome by high-throughput sequencing.* Nat Genet, 2008. **40**(12): p. 1413-5.

2.	Wang, E.T., et al., *Alternative isoform regulation in human tissue transcriptomes.* Nature, 2008. **456**(7221): p. 470-6.

3.	Kelemen, O., et al., *Function of alternative splicing.* Gene, 2013. **514**(1): p. 1-30.

4.	El Marabti, E. and I. Younis, *The Cancer Spliceome: Reprograming of Alternative Splicing in Cancer.* Front Mol Biosci, 2018. **5**: p. 80.

5.	Climente-Gonzalez, H., et al., *The Functional Impact of Alternative Splicing in Cancer.* Cell Rep, 2017. **20**(9): p. 2215-2226.

6.	David, C.J. and J.L. Manley, *Alternative pre-mRNA splicing regulation in cancer: pathways and programs unhinged.* Genes Dev, 2010. **24**(21): p. 2343-64.

7.	Kim, E., A. Goren, and G. Ast, *Insights into the connection between cancer and alternative splicing.* Trends Genet, 2008. **24**(1): p. 7-10.

8.	Lee, S.C. and O. Abdel-Wahab, *Therapeutic targeting of splicing in cancer.* Nat Med, 2016. **22**(9): p. 976-86.

9.	Ghigna, C., et al., *Cell motility is controlled by SF2/ASF through alternative splicing of the Ron protooncogene.* Mol Cell, 2005. **20**(6): p. 881-90.

10.	Cohen-Eliav, M., et al., *The splicing factor SRSF6 is amplified and is an oncoprotein in lung and colon cancers.* J Pathol, 2013. **229**(4): p. 630-9.

11.	Guo, X., et al., *Exon array analysis reveals neuroblastoma tumors have distinct alternative splicing patterns according to stage and MYCN amplification status.* BMC Med Genomics, 2011. **4**: p. 35.

12.	Hui, L., et al., *Identification of alternatively spliced mRNA variants related to cancers by genome-wide ESTs alignment.* Oncogene, 2004. **23**(17): p. 3013-23.

13.	Relogio, A., et al., *Alternative splicing microarrays reveal functional expression of neuron-specific regulators in Hodgkin lymphoma cells.* J Biol Chem, 2005. **280**(6): p. 4779-84.

14.	Thorsen, K., et al., *Alternative splicing in colon, bladder, and prostate cancer identified by exon array analysis.* Mol Cell Proteomics, 2008. **7**(7): p. 1214-24.

15.	Xu, Q. and C. Lee, *Discovery of novel splice forms and functional analysis of cancer-specific alternative splicing in human expressed sequences.* Nucleic Acids Res, 2003. **31**(19): p. 5635-43.

16.	Castellana, S., et al., *Systematic Analysis of Mouse Genome Reveals Distinct Evolutionary and Functional Properties Among Circadian and Ultradian Genes.* Front Physiol, 2018. **9**: p. 1178.

17.	Torres, M., et al., *Circadian processes in the RNA life cycle.* Wiley Interdiscip Rev RNA, 2018.

18.	Lee, K.H., et al., *Rhythmic interaction between Period1 mRNA and hnRNP Q leads to circadian time-dependent translation.* Mol Cell Biol, 2012. **32**(3): p. 717-28.

19.	Morf, J., et al., *Cold-inducible RNA-binding protein modulates circadian gene expression posttranscriptionally.* Science, 2012. **338**(6105): p. 379-83.

20.	Kojima, S., et al., *LARK activates posttranscriptional expression of an essential mammalian clock protein, PERIOD1.* Proc Natl Acad Sci U S A, 2007. **104**(6): p. 1859-64.

21.	Lopez-Urrutia, E., et al., *Alternative splicing regulation in tumor necrosis factor-mediated inflammation.* Oncol Lett, 2017. **14**(5): p. 5114-5120.

22.	Rothe, J., et al., *Tumor necrosis factor receptors--structure and function.* Immunol Res, 1992. **11**(2): p. 81-90.

23.	Tracey, K.J. and A. Cerami, *Tumor necrosis factor, other cytokines and disease.* Annu Rev Cell Biol, 1993. **9**: p. 317-43.

24.	Balkwill, F., *Tumour necrosis factor and cancer.* Nat Rev Cancer, 2009. **9**(5): p. 361-71.

25.	Wu, Y. and B.P. Zhou, *TNF-alpha/NF-kappaB/Snail pathway in cancer cell migration and invasion.* Br J Cancer, 2010. **102**(4): p. 639-44.

26.	Johnston, D.A., B. Dong, and C.C. Hughes, *TNF induction of jagged-1 in endothelial cells is NFkappaB-dependent.* Gene, 2009. **435**(1-2): p. 36-44.

27.	El-Athman, R., L. Fuhr, and A. Relogio, *A Systems-Level Analysis Reveals Circadian Regulation of Splicing in Colorectal Cancer.* EBioMedicine, 2018. **33**: p. 68-81.

28.	Ye, Y., et al., *The Genomic Landscape and Pharmacogenomic Interactions of Clock Genes in Cancer Chronotherapy.* Cell Syst, 2018. **6**(3): p. 314-328 e2.

29.	Dvinge, H., et al., *RNA splicing factors as oncoproteins and tumour suppressors.* Nat Rev Cancer, 2016. **16**(7): p. 413-30.

30.	Guo, W., et al., *TSIS: an R package to infer alternative splicing isoform switches for time-series data.* Bioinformatics, 2017. **33**(20): p. 3308-3310.

31.	Vitting-Seerup, K. and A. Sandelin, *The Landscape of Isoform Switches in Human Cancers.* Mol Cancer Res, 2017. **15**(9): p. 1206-1220.

32.	Ferrara, N., et al., *Targeting VEGF-A to treat cancer and age-related macular degeneration.* Annu Rev Med, 2007. **58**: p. 491-504.

33.	Gara, R.K., et al., *Slit/Robo pathway: a promising therapeutic target for cancer.* Drug Discov Today, 2015. **20**(1): p. 156-64.

34.	Ornitz, D.M. and N. Itoh, *The Fibroblast Growth Factor signaling pathway.* Wiley Interdiscip Rev Dev Biol, 2015. **4**(3): p. 215-66.

35.	Li, Y., et al., *HNRNPH1 is required for rhabdomyosarcoma cell growth and survival.* Oncogenesis, 2018. **7**(1): p. 9.

36.	Sun, Y.L., et al., *Protein and gene expression characteristics of heterogeneous nuclear ribonucleoprotein H1 in esophageal squamous cell carcinoma.* World J Gastroenterol, 2016. **22**(32): p. 7322-31.

37.	Gautrey, H., et al., *SRSF3 and hnRNP H1 regulate a splicing hotspot of HER2 in breast cancer cells.* RNA Biol, 2015. **12**(10): p. 1139-51.

38.	Marriott, A.S., et al., *NUDT2 Disruption Elevates Diadenosine Tetraphosphate (Ap4A) and Down-Regulates Immune Response and Cancer Promotion Genes.* PLoS One, 2016. **11**(5): p. e0154674.

39.	Postel, E.H., et al., *Human c-myc transcription factor PuF identified as nm23-H2 nucleoside diphosphate kinase, a candidate suppressor of tumor metastasis.* Science, 1993. **261**(5120): p. 478-80.

40.	Herrlich, P., et al., *CD44 acts both as a growth- and invasiveness-promoting molecule and as a tumor-suppressing cofactor.* Ann N Y Acad Sci, 2000. **910**: p. 106-18; discussion 118-20.

41.	Wang, L., et al., *CPAT: Coding-Potential Assessment Tool using an alignment-free logistic regression model.* Nucleic Acids Res, 2013. **41**(6): p. e74.

42.	Finn, R.D., et al., *Pfam: the protein families database.* Nucleic Acids Res, 2014. **42**(Database issue): p. D222-30.

43.	Kar, A., et al., *Metastases suppressor NME2 associates with telomere ends and telomerase and reduces telomerase activity within cells.* Nucleic Acids Res, 2012. **40**(6): p. 2554-65.

44.	Eberth, S., et al., *Epigenetic regulation of CD44 in Hodgkin and non-Hodgkin lymphoma.* BMC Cancer, 2010. **10**: p. 517.

45.	Yan, Y., X. Zuo, and D. Wei, *Concise Review: Emerging Role of CD44 in Cancer Stem Cells: A Promising Biomarker and Therapeutic Target.* Stem Cells Transl Med, 2015. **4**(9): p. 1033-43.

46.	Liu, J. and G. Jiang, *CD44 and hematologic malignancies.* Cell Mol Immunol, 2006. **3**(5): p. 359-65.

47.	Francalanci, P., et al., *CD44-v6 expression in smooth muscle cells in the postnatal remodeling process of ductus arteriosus.* Am J Cardiol, 2006. **97**(7): p. 1056-9.

48.	Zhou, G., et al., *Protein phosphatase 4 is involved in tumor necrosis factor-alpha-induced activation of c-Jun N-terminal kinase.* J Biol Chem, 2002. **277**(8): p. 6391-8.

49.	Hsu, P.L. and S.M. Hsu, *Abundance of heat shock proteins (hsp89, hsp60, and hsp27) in malignant cells of Hodgkin's disease.* Cancer Res, 1998. **58**(23): p. 5507-13.

50.	Wang, T., et al., *Knockdown of the differentially expressed gene TNFRSF12A inhibits hepatocellular carcinoma cell proliferation and migration in vitro.* Mol Med Rep, 2017. **15**(3): p. 1172-1178.

51.	Wiley, S.R., et al., *A novel TNF receptor family member binds TWEAK and is implicated in angiogenesis.* Immunity, 2001. **15**(5): p. 837-46.

52.	Kato, T., Jr., et al., *Negative regulation of constitutive NF-kappaB and JNK signaling by PKN1-mediated phosphorylation of TRAF1.* Genes Cells, 2008. **13**(5): p. 509-20.

53.	Lavorgna, A., et al., *TNF receptor-associated factor 1 is a positive regulator of the NF-kappaB alternative pathway.* Mol Immunol, 2009. **46**(16): p. 3278-82.

54.	Ikeda, F., et al., *SHARPIN forms a linear ubiquitin ligase complex regulating NF-kappaB activity and apoptosis.* Nature, 2011. **471**(7340): p. 637-41.

55.	Zhang, Y., et al., *Analysis of NFKB2mediated regulation of mechanisms underlying the development of Hodgkin's lymphoma.* Mol Med Rep, 2018. **17**(6): p. 8129-8136.

56.	Hong, H.K., et al., *Requirement for NF-kappaB in maintenance of molecular and behavioral circadian rhythms in mice.* Genes Dev, 2018. **32**(21-22): p. 1367-1379.

57.	Leeman, J.R. and T.D. Gilmore, *Alternative splicing in the NF-kappaB signaling pathway.* Gene, 2008. **423**(2): p. 97-107.

58.	Yu, G., et al., *clusterProfiler: an R package for comparing biological themes among gene clusters.* OMICS, 2012. **16**(5): p. 284-7.
